# Ochratoxin A and Clear Cell Renal Cell Carcinoma: Exploring Potential Molecular Links Through Network Toxicology and Machine Learning

**DOI:** 10.3390/ijms27072971

**Published:** 2026-03-25

**Authors:** Chenjie Huang, Lulu Wei, Wenqi Yuan, Yaohong Lu, Ziyou Yan, Gedi Zhang

**Affiliations:** School of Clinical Medicine, Jiangxi University of Chinese Medicine, Nanchang 330004, China; huangchenjie@jxutcm.edu.cn (C.H.); 18702534935@163.com (L.W.); m15970290352@163.com (W.Y.); m13978171592@163.com (Y.L.)

**Keywords:** clear cell renal cell carcinoma, Ochratoxin A, network toxicology, machine learning, molecular docking, molecular mechanism

## Abstract

Ochratoxin A (OTA), a prevalent food contaminant, is closely linked to the development of various cancers, including clear cell renal cell carcinoma (ccRCC). However, the potential mechanisms remain to be explored. In this study, we employed network toxicology, machine learning, and molecular docking techniques to systematically investigate the potential molecular mechanisms underlying OTA-associated ccRCC. We normalized transcriptional data from two Gene Expression Omnibus (GEO) datasets and analyzed it using differential expression analysis and weighted gene co-expression network analysis (WGCNA), identifying 3224 ccRCC-associated target genes. These were intersected with 232 predicted OTA target genes, yielding a total of 56 overlapping targets. The results of Gene Ontology (GO) and Kyoto Encyclopedia of Genes and Genomes (KEGG) analyses indicated that these targets were primarily enriched in critical biological processes, including extracellular matrix remodeling, immune microenvironment regulation, signaling pathway transduction, cellular metabolism, and protein homeostasis. Machine learning analysis identified “glmBoost + RF” (a sequential combination of feature selection and classifier) as the optimal model, from which nine key genes were extracted. SHapley Additive exPlanations (SHAP) analysis revealed five core genes (*IGFBP3*, *ITGA5*, *PYGL*, *SLC22A8*, *LTB4R*), with *IGFBP3* and *ITGA5* serving as the principal driver genes of the model. Validation of the model’s diagnostic efficacy and single-cell transcriptome analysis indicated that the core genes exhibited significant differential expression patterns, cell-type-specific expression characteristics, and high independent diagnostic efficacy. Molecular docking analyses predicted stable interactions between OTA and the core target proteins. These findings suggest potential molecular links between OTA exposure and ccRCC, providing a foundation for hypothesis generation and future experimental validation.

## 1. Introduction

Ochratoxin A (OTA) is a mycotoxin produced by fungi of the genera Aspergillus and Penicillium. It can be detected in various agricultural products, including grains, nuts, wine, coffee, cheese, and processed meats, and is prevalent throughout all stages of food processing, from raw materials to finished products [1]. Due to its primary exposure to humans through dietary intake, along with its strong toxicity and high stability, OTA has emerged as a significant food contaminant globally, posing serious threats to human health and food safety [2]. Statistics indicate that OTA contamination is widespread across different geographic regions, with levels in various foods in some countries reaching as high as 139.20 μg/kg [3]. Additionally, approximately 25% or more of the world’s agricultural products are contaminated annually by mycotoxins such as OTA. This leads to significant economic losses due to issues like agricultural food destruction, feed bans, and meat product recalls [4,5]. In 1993, the International Agency for Research on Cancer (IARC) classified OTA as a group 2B carcinogen (possibly carcinogenic to humans) [6]. In 1995, the Joint FAO/WHO Expert Committee on Food Additives (JECFA) further established a provisional tolerable daily intake (PTDI) for OTA of 14 ng/kg body weight [7]. Available studies indicate that OTA exhibits substantial nephrotoxicity and carcinogenicity in various animal models, and its mechanism of action involves multiple pathways, including the induction of oxidative stress, Deoxyribonucleic acid (DNA) damage, disruption of the cell cycle, interference with cell signaling, and alterations in epigenetics [8]. Despite the clear demonstration of OTA’s carcinogenicity, existing research is limited by challenges such as difficulties in exposure assessment, inadequate control of confounding factors, pronounced population heterogeneity, and inconsistent conclusions. These limitations have hindered a comprehensive understanding of the specific carcinogenic mechanisms at the molecular level. Consequently, it is crucial to further investigate the toxicological properties of OTA, particularly to elucidate its molecular mechanisms of carcinogenicity, which holds significant implications for human health and food safety.

Renal cell carcinoma (RCC) is a prevalent malignant tumor of the urinary system, representing approximately 2% of all cancer diagnoses and deaths globally [9]. The Global Cancer Observatory (GLOBOCAN) 2022 global cancer statistics report indicates that RCC ranks 14th in incidence and 16th in mortality among all malignant tumors [10]. Clear cell renal cell carcinoma (ccRCC) is the most common subtype of RCC, comprising about 85% of RCC cases and accounting for 75% of RCC-related deaths; it is the primary pathological type contributing to the morbidity and mortality rates associated with RCC [11,12]. ccRCCs arise from renal tubular epithelial cells and exhibit significant histological heterogeneity, including variations in tumor cell morphology and structural diversity, as well as molecular heterogeneity characterized by gene mutations and differences in expression profiles [13]. Studies indicate that early-stage ccRCC is highly insidious, with approximately 30% of patients presenting with metastasis at diagnosis and a high risk of distant organ spread. Advanced disease commonly manifests with the triad of flank pain, hematuria, and abdominal mass. Unfortunately, the overall prognosis for ccRCC remains poor, as most patients are diagnosed at intermediate or advanced stages of the disease [14]. The therapeutic approaches for ccRCC encompass local interventions, such as surgical resection or ablation, as well as systemic treatments, including immunotherapy and targeted therapies [15]. Although these strategies have demonstrated efficacy in certain patients, the overall effectiveness remains limited for the majority due to the tumor’s high metastatic potential, recurrence tendencies, and drug resistance. Recent research indicates that, in addition to intrinsic factors like genetic mutations, genomic instability, and epigenetic inheritance [16], environmental influences—such as exposure to microplastics and fungal toxins—may also contribute to the development of ccRCC [17,18]. Notably, the mycotoxin OTA, a prevalent food contaminant, has been established as nephrotoxic and carcinogenic; however, the existence of a direct causal relationship between OTA and ccRCC, along with the underlying molecular mechanisms, remains uncertain. Limited in vitro studies have reported that OTA exposure leads to the upregulation of WNT1 inducible signaling pathway protein 1 antisense RNA 1 (WISP1-AS1) expression in human proximal tubular cells, and this upregulation of the long non-coding Ribonucleic acid (RNA)—frequently associated with the malignant transformation of ccRCC cells and exhibiting a significant aberrant expression pattern—suggests a potential link between OTA and ccRCC [19]. However, a clinical study by Fahmy et al. [20] indicated that while OTA can be detected in the serum of patients with RCC, including ccRCC, its concentration remains low, ranging from 0.004 to 0.25 ng/mL. Additionally, immunohistochemical staining revealed that OTA signals were absent in all RCC tissues, suggesting a lack of direct evidence for OTA-induced RCC in humans. Consequently, the conclusion regarding whether OTA exposure contributes to the development of ccRCC remains contentious. The kidney, as a vital organ for metabolic detoxification and excretion, is particularly susceptible to damage from exogenous compounds and their toxic metabolites. OTA and its metabolites may affect renal tubular epithelial cells of ccRCC origin by inducing oxidative stress, DNA damage, and other pathways. However, comprehensive studies investigating these molecular mechanisms are still lacking. Therefore, further systematic research at both toxicological and molecular biological levels is essential to elucidate the specific mechanisms by which OTA exposure influences the development of ccRCC.

Traditional toxicology studies primarily focus on a single target or pathway, which hinders a comprehensive understanding of the complex molecular mechanisms underlying OTA-associated ccRCC. To address this limitation, the present study innovatively integrates network toxicology, machine learning, and molecular docking technology to establish a multilevel research framework. First, we constructed the “compound-target-disease” association network using network toxicology, systematically screening the intersecting targets and relevant pathways associated with OTA and ccRCC. Next, we employed machine learning methods to prioritize key pivotal genes and assessed model performance using the SHapley Additive exPlanations (SHAP) interpretable method, selecting target genes with greater contributions as core genes. We then validated the diagnostic efficacy of these core genes and conducted single-cell transcriptome analysis. Finally, molecular docking techniques were utilized to predict the interaction patterns between OTA and the core target proteins, as well as to evaluate their binding stability (Appendix A provides a comprehensive flowchart illustrating the entire analysis process). This analytical process establishes a comprehensive research loop of “target prediction-gene screening-multiple validation,” which aids in systematically elucidating the molecular mechanisms of OTA-associated ccRCC development and provides a scientific foundation for the development of ccRCC-targeted drugs and the toxicological evaluation of OTA.

## 2. Results

### 2.1. Identification of OTA Target Genes

**Figure 1:**
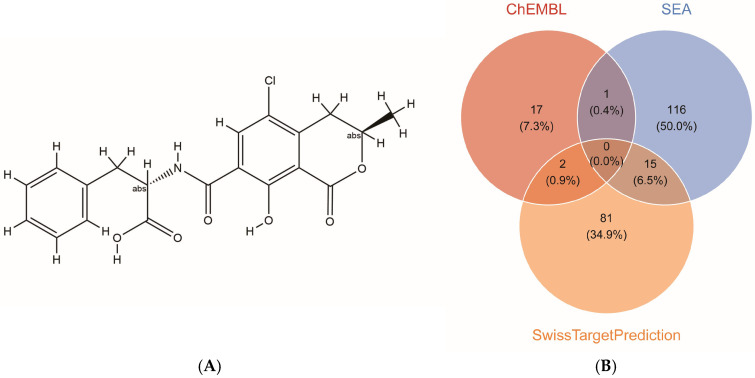
Identification of Ochratoxin A (OTA) target genes. (**A**) Chemical structure diagram of OTA. (**B**) Venn diagram illustrating the integration results from three distinct databases—namely, ChEMBL, SwissTargetPrediction, and SEA—used to predict potential target genes of OTA (*n* = 232).

The molecular structure of OTA was retrieved from the PubChem database (Figure 1A). Its potential targets were systematically predicted using an integrative approach that combined three complementary databases: ChEMBL, SEA, and SwissTargetPrediction. After merging and deduplicating the prediction results from various databases, 232 potential target genes for OTAs were ultimately identified (Figure 1B).

### 2.2. Identification of ccRCC-Related Target Genes

**Figure 2:**
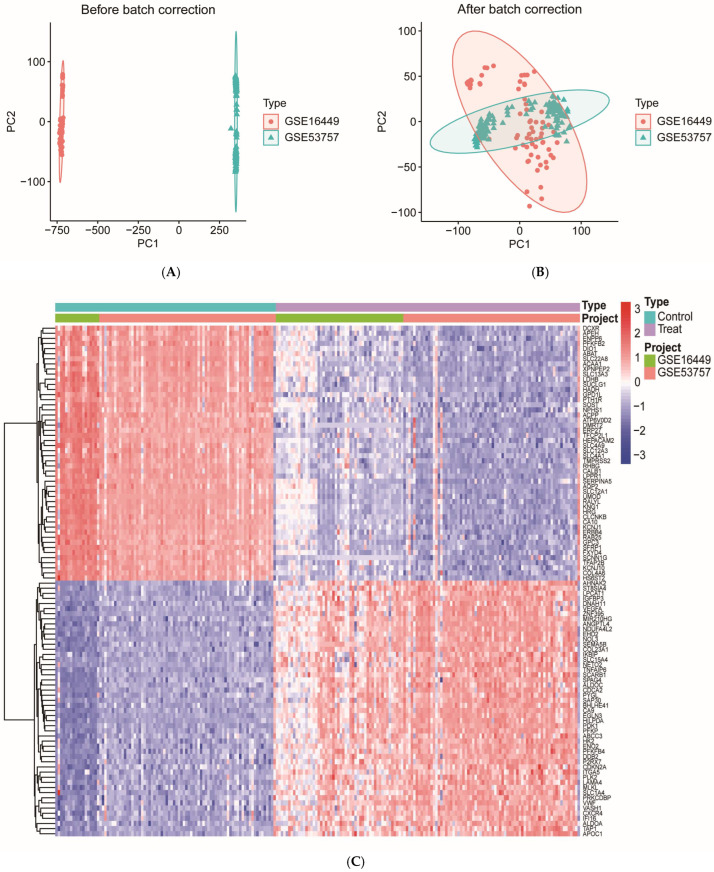

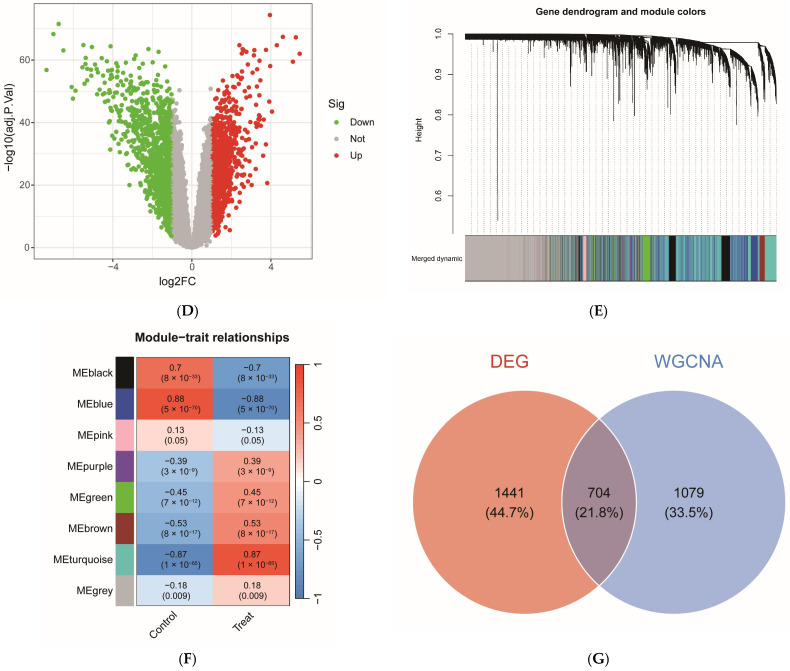
Screening of clear cell renal cell carcinoma (ccRCC)-related target genes. (**A**) The principal component analysis (PCA) plots prior to batch correction indicated a clear spatial separation of sample points from the two ccRCC transcriptome datasets (GSE16449 and GSE53757) obtained from the Gene Expression Omnibus (GEO) database, suggesting the presence of a batch effect in the data. (**B**) The batch-corrected PCA plot illustrates effective fusion of the sample points from both datasets, indicating successful control of the batch effect. (**C**) A clustered heatmap displays the expression patterns of the 50 most significantly up-regulated and down-regulated genes in 124 tumor samples compared to 90 normal samples. Gene expression values were normalized using z-scores, with red indicating high expression and blue indicating low expression. Hierarchical clustering analyses were conducted on both rows (genes) and columns (samples). (**D**) The volcano plot depicts the distribution characteristics of 2145 differentially expressed genes identified using thresholds of false discovery rate (FDR)-corrected *p* < 0.05 and |log_2_FC| > 0.585, with log_2_ fold change (tumor vs. normal) plotted on the *x*-axis and −log10 (*p*-value) on the *y*-axis. For visualization clarity, gene classification criteria are as follows: up-regulation (red): log_2_FC > 1 and *p* < 0.05; down-regulation (green): log_2_FC < −1 and *p* < 0.05; and no significant difference (gray): |log_2_FC| ≤ 1 or *p* ≥ 0.05. The separation of red and green dots reflects the mutually exclusive nature of positive and negative fold changes. (**E**) The gene clustering tree and module color map derived from weighted gene co-expression network analysis (WGCNA) illustrate the results of hierarchical clustering based on co-expression. The module color map below delineates distinct gene modules, categorizing all gene systems into eight independent modules characterized by co-expression. (**F**) The module–trait association heatmap reveals the degree of association between the modules identified by WGCNA and sample traits, with correlation coefficients and *p*-values indicated in each box. This suggests that multiple modules exhibit significant associations with ccRCC phenotypes (*p* < 0.05). (**G**) The Venn diagram illustrates differentially expressed genes (red, *n* = 2145) that are significantly associated with ccRCC alongside WGCNA module genes (blue, *n* = 1783). The intersection of the two sets contains 704 shared target genes, and the percentages in the figure represent the proportion of genes from each region relative to the total union (*n* = 3224).

To mitigate batch effect interference, the two ccRCC datasets (GSE16449, GSE53757) were merged in this study, and the gene expression matrices underwent quantile normalization followed by log_2_ transformation. Principal component analysis (PCA) results indicated an improved distribution of the normalized cohort data, revealing a clearer clustering pattern and demonstrating effective control of the batch effect (Figure 2A,B). Gene differential expression analysis identified 2145 differentially expressed genes (DEGs) that exhibited significant alterations in ccRCC, using thresholds of false discovery rate (FDR)-corrected *p* < 0.05 and |log_2_FC| > 0.585, with their expression changes visualized through clustering heatmaps and volcano plots (Figure 2C,D). Additionally, weighted gene co-expression network analysis (WGCNA) was employed to construct gene co-expression networks. The optimal soft-threshold power (β = 9) was calculated to ensure that the network adhered to scale-free topology. Subsequently, a topology overlap matrix was constructed, and hierarchical clustering was performed based on this parameter, resulting in the delineation of eight distinct gene modules (Figure 2E), each represented by different color markers. Module–trait association analysis revealed that the blue module was most significantly correlated with ccRCC (|R| > 0.5, *p* < 0.05, Figure 2F). From this module, 1783 genes were extracted based on module membership [module membership (kME) > 0.8] and gene significance [gene significance (GS) > 0.2]. The union of the 2145 DEGs and the 1783 module genes was formed, yielding a total of 3224 ccRCC-associated target genes, with 704 genes overlapping between the two sets (Figure 2G). To assess the robustness of these findings, we performed a sensitivity analysis by varying the soft-threshold power (β = 8–10) and module merging height (mergeCutHeight = 0.20–0.30). Across all tested parameter combinations, the union-based overlap between ccRCC-associated genes and predicted OTA targets remained stable (43–45 genes), and all five core genes subsequently identified by SHAP analysis were consistently present (Supplementary Materials Table S1).

### 2.3. Identification and Functional Enrichment Analysis of OTA and ccRCC Intersection Targets

**Figure 3:**
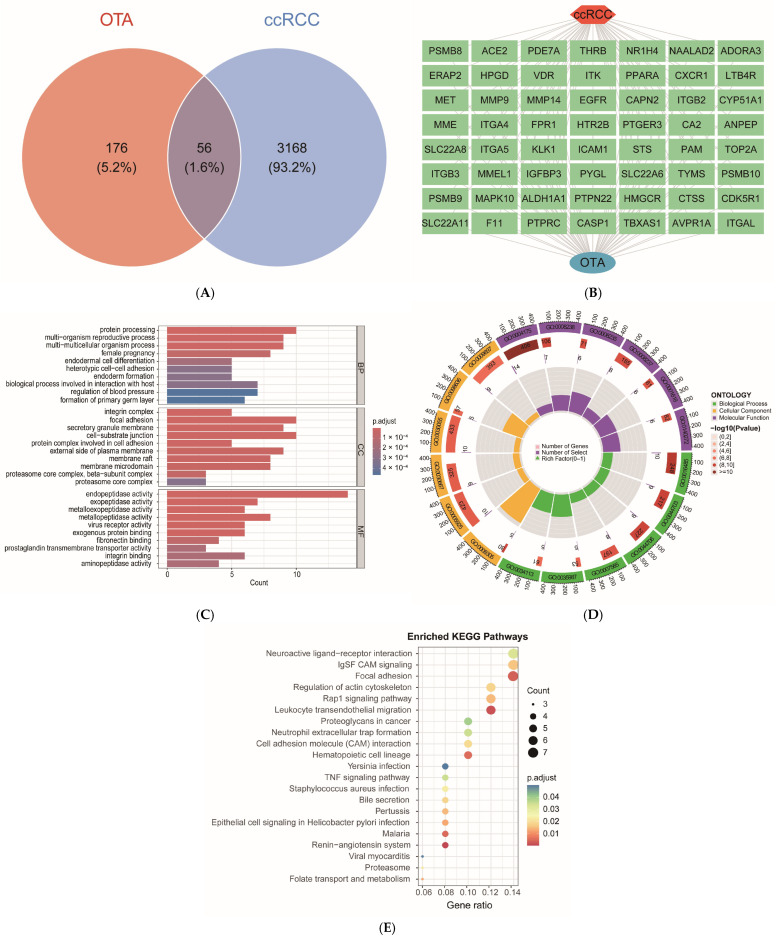

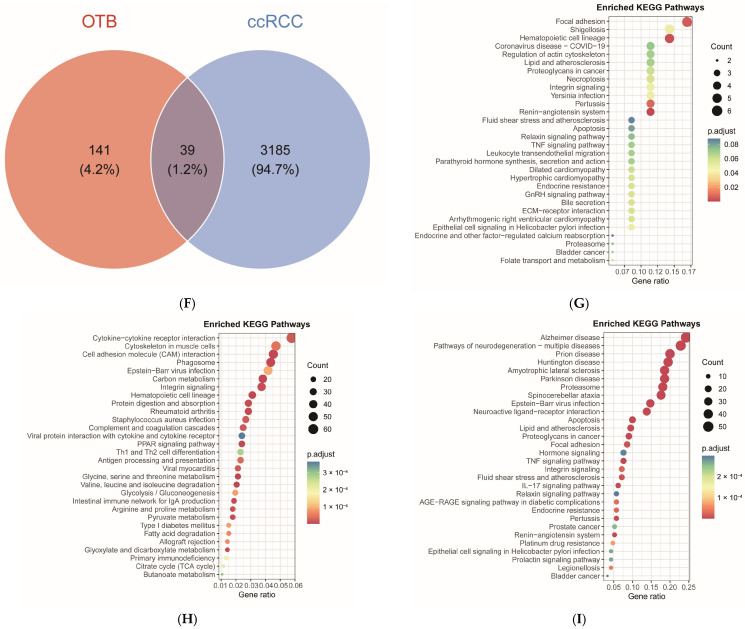
Identification and functional enrichment analysis of intersecting targets related to OTA and ccRCC. (**A**) A Venn diagram illustrates the intersection between genes associated with OTA exposure (red, *n* = 232) and those related to ccRCC (blue, *n* = 3224), revealing a total of 56 shared target genes, which constitutes 1.6% of the overall gene set (*n* = 3400). The diagram displays the number of genes, while the percentages indicate the proportion of genes in each section relative to the total. (**B**) The OTA–ccRCC association network graph depicts the regulatory network of compound → gene → disease associations. In this graph, blue nodes represent compounds, green nodes signify genes, red nodes denote diseases, and connecting lines illustrate predicted interactions. (**C**) A Gene Ontology (GO) bar graph presents the annotation of the intersecting genes across biological process (BP), cellular component (CC), and molecular function (MF). The *x*-axis indicates the gene counts for each entry, while the *y*-axis lists the names of the GO function entries. The color gradient of the bars reflects the corrected *p*-value, with deeper red hues indicating greater enrichment significance. (**D**) The GO circle plot illustrates the enrichment of intersecting genes across various GO categories. The outermost scale represents the total number of genes on a logarithmic scale with a base of 10. The first circle denotes the pathway number and classification. The length of the rectangles in the second circle corresponds to the number of genes within each GO entry, with darker colors indicating greater enrichment significance. The length of the rectangles in the third circle reflects the number of genes overlapping with the respective GO entry in the input gene set. The fourth circle depicts the ratio of the number of genes in the third circle to the corresponding number of genes in the second circle. (**E**) The Kyoto Encyclopedia of Genes and Genomes (KEGG) bubble map displays the enrichment pathways of intersecting genes. The *x*-axis represents the gene ratio, calculated as the number of genes associated with a specific KEGG entry in the target gene set divided by the total number of genes in the KEGG entries. The *y*-axis lists the KEGG pathway names. The size of the bubbles indicates the number of genes counted, while the color gradient represents the corrected *p*-value, with a redder hue signifying higher enrichment significance. (**F**) A Venn diagram illustrates the intersection between genes associated with OTA exposure (red, *n* = 180) and those related to ccRCC (blue, *n* = 3224), revealing a total of 39 shared target genes, which constitutes 1.2% of the overall gene set (*n* = 3365). The diagram displays the number of genes, while the percentages indicate the proportion of genes in each section relative to the total. (**G**) The KEGG bubble map displays the enrichment pathways of Ochratoxin B (OTB)–ccRCC genes. (**H**) The KEGG bubble map displays the enrichment pathways of ccRCC differentially expressed genes (DEGs). (**I**) The KEGG bubble map displays the enrichment pathways of OTA genes.

The 232 potential target genes associated with OTA were intersected with 3224 genes related to ccRCC, resulting in the identification of 56 overlapping target genes (Figure 3A,B). These intersected genes were subjected to Gene Ontology (GO) function and Kyoto Encyclopedia of Genes and Genomes (KEGG) pathway enrichment analyses. The GO analysis revealed that, within the biological process (BP) category, the target genes were predominantly enriched in protein processing, multicellular biological processes, endoderm formation, and host interactions. In the cellular component (CC) category, the target genes were primarily associated with structures such as the integrin complex, adhesion plaques, secretory granule membranes, and the proteasomal core complex. Regarding molecular function (MF), the genes exhibited significant enrichment in endopeptidase/exopeptidase activity, metallopeptidase activity, viral receptor activity, exogenous protein binding, and integrin binding (Figure 3C,D). Furthermore, KEGG pathway enrichment analysis further indicates that these target genes are extensively involved in metabolic regulation (e.g., folate transport and metabolism, bile secretion), cancer-related signaling [e.g., neuroactive ligand-receptor interactions, Ras-related protein 1 (Rap1), tumor necrosis factor (TNF), immunoglobulin superfamily cell adhesion molecule (IgSF CAM) signaling pathways], immune and infection responses (e.g., leukocyte transendothelial migration, neutrophil extracellular trap formation, and pathways related to viral, bacterial, and parasitic infections), and cell adhesion and cytoskeletal regulation (e.g., focal adhesions, cell adhesion molecule interactions, hematopoietic cell lineage) (Figure 3E). These findings suggest that the potential targets of OTA are extensively implicated in various aspects of ccRCC development. Potential mechanisms may involve the modulation of extracellular matrix (ECM) remodeling, which is mediated by peptidase activity and integrin binding, regulation of the tumor immune microenvironment, and key signaling pathways such as TNF and Rap1. Additionally, these targets may influence cellular metabolism and protein homeostasis, including proteasome function, among other processes. This study establishes a crucial foundation for elucidating the molecular mechanisms underlying OTA-associated ccRCC and for developing targeted interventions. The detailed information for each gene set in the Venn diagram is provided in Supplementary Materials Table S2.

To evaluate whether the observed OTA–ccRCC association is specific to OTA or reflects generic mycotoxin properties, we performed a negative control analysis using Ochratoxin B (OTB), a structural analog lacking the chlorine atom. The target prediction pipeline for OTB yielded 180 predicted genes, of which only 39 overlapped with ccRCC-associated genes—substantially fewer than the 56 genes identified for OTA (Supplementary Materials Table S3, Figure 3F). KEGG enrichment analysis of the OTB–ccRCC overlapping genes revealed both shared and distinct pathway enrichment patterns compared to OTA. While OTB also enriched some cancer-related pathways (e.g., focal adhesion, TNF signaling, proteoglycans in cancer), critical pathways implicated in ccRCC pathogenesis—such as Rap1 signaling, cell adhesion molecule (CAM) interactions, and IgSF CAM signaling—were uniquely enriched in the OTA–ccRCC overlap and completely absent in the OTB analysis. Moreover, for pathways common to both compounds (e.g., focal adhesion, TNF signaling), the enrichment significance was substantially stronger for OTA. Notably, OTB uniquely enriched ECM–receptor interaction—a pathway not significantly enriched by OTA—further highlighting the distinct biological mechanisms of the two analogs (Figure 3G). These results suggest that the OTA–ccRCC association is highly specific and may be attributable to structural features unique to OTA, particularly the chlorine atom.

To further assess the specificity of functional enrichment in OTA–ccRCC overlapping genes, we compared their KEGG enrichment profiles with those of ccRCC DEGs and OTA targets alone (Figure 3H,I). As illustrated, several pathways were shared across multiple gene sets (e.g., focal adhesion, TNF signaling, proteoglycans in cancer). Critically, pathways linked to ccRCC pathogenesis—including the Rap1 signaling pathway and IgSF CAM signaling pathway—were uniquely enriched in the OTA–ccRCC overlap but absent in both ccRCC DEGs alone and OTA targets. Additionally, CAM interactions were enriched in both the OTA–ccRCC overlap and ccRCC DEGs, yet notably absent in OTA targets alone, suggesting that OTA may specifically enhance these adhesion-related mechanisms beyond general ccRCC biology. These comparative analyses suggest that OTA–ccRCC overlapping genes capture a distinct set of cancer-relevant pathways—particularly those involved in cell adhesion, migration, and signaling—rather than merely reflecting generic ccRCC biology or non-specific OTA toxicity.

### 2.4. Identification of OTA-Associated ccRCC Core Genes and Validation of Diagnostic Efficacy

**Figure 4:**
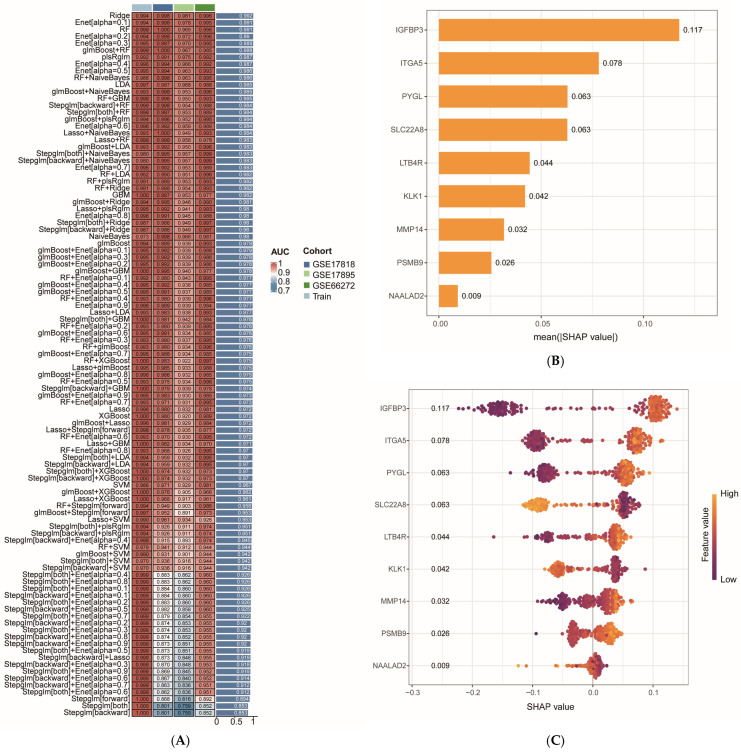

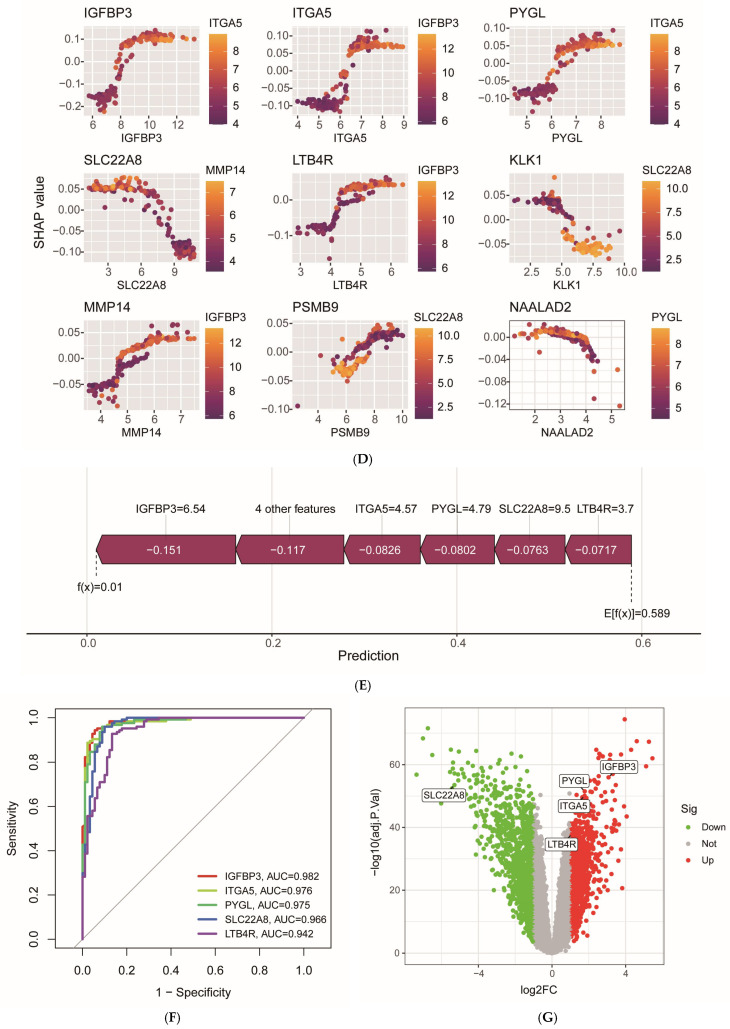

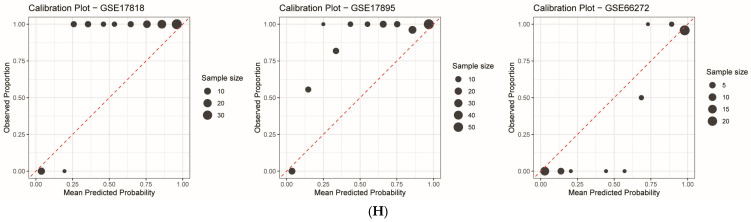
Machine learning-based screening of core genes and their validation. (**A**) The performance heatmap of the machine learning models displays the Area Under the Curve (AUC) values of 113 prediction models in the training set (5-fold cross-validation) and three independent validation sets (GSE17818, GSE17895, GSE66272). The left column lists the model names, while the right column presents the corresponding AUC values, with redder colors indicating larger AUC values. Different colored squares in the top column represent various cohort sources. (**B**) The SHapley Additive exPlanations (SHAP) feature importance histogram illustrates the top 15 genes ranked by their average absolute SHAP values, where longer bars indicate a greater average contribution of each gene to the model’s prediction results. (**C**) The SHAP summary plot depicts the distribution of SHAP values for each gene across all samples. The *x*-axis represents the SHAP value, while the *y*-axis denotes the gene names. Each point corresponds to an individual sample, and the color indicates the expression level of the gene for that sample (Feature value), with purple representing low expression and orange indicating high expression. The width of the plot reflects the density of samples within a specific SHAP value interval. (**D**) The SHAP-dependent scatterplot illustrates the nonlinear relationship between the expression levels of key genes (*x*-axis) and their contributions to the model output (SHAP value, *y*-axis). This visualization reveals the interaction thresholds and synergistic effects among the key genes, while the gradient color transition from purple (low expression) to orange (high expression) indicates the varying effects of gene expression. (**E**) The force-directed plot depicts how all features in a single sample influence the model’s baseline predicted value, ultimately leading to the final predicted output for that sample. Features contributing positively (indicating elevated risk) are represented in orange, whereas features contributing negatively (indicating reduced risk) are shown in purple. (**F**) The diagnostic ROC curves for the core genes demonstrate the capacity of the five core genes (*IGFBP3*, *ITGA5*, *PYGL*, *SLC22A8*, *LTB4R*) to independently distinguish between ccRCC tissues and normal tissues. The *x*-axis represents the false-positive rate, the *y*-axis indicates sensitivity, and the dashed line denotes the baseline performance of the stochastic model (AUC = 0.5). The AUC value reflects predictive accuracy, with values closer to 1 indicating stronger predictive ability. (**G**) The volcano plot of differentially expressed genes reveals the transcriptomic differences between ccRCC and normal tissues. The *x*-axis represents log_2_FC, and the *y*-axis represents −log10(*p*-value). Red and green dots signify significantly up-regulated and down-regulated genes, respectively (FDR-corrected *p* < 0.05 and |log_2_(FC)| > 0.585), with the five core genes specifically labeled. (**H**) Calibration plots for the glmBoost + RF model in three external validation cohorts. The *x*-axis shows the mean predicted probability of ccRCC (range 0–1), and the *y*-axis shows the observed proportion of ccRCC in each bin. Points represent groups of samples with similar predicted probabilities; dot size reflects the number of samples in each group (*n* = 10, 20, or 30). The red dashed line indicates perfect calibration (observed = predicted). Points close to this line indicate good agreement between predictions and observations, demonstrating satisfactory calibration performance with no substantial systematic bias.

To identify the core driver genes associated with OTA-associated ccRCC, we analyzed 56 intersecting target genes using machine learning techniques and constructed 113 predictive models by integrating various algorithms. The performance of these models was assessed through the average Area Under the Curve (AUC) value, revealing that the combined “glmBoost + RF” (a sequential combination of feature selection and classifier) model exhibited outstanding performance during both the training and validation phases (Figure 4A). The “Generalized Linear Model Boosting (glmBoost)” algorithm initially identified the most informative gene features, followed by the “Random Forest (RF)” algorithm, which constructed the final prediction model. This “glmBoost + RF” designation indicates a sequential combination of feature selection and classifier, rather than an ensemble or stacked model. Utilizing this model, we extracted nine key genes (*IGFBP3*, *ITGA5*, *SLC22A8*, *PYGL*, *MMP14*, *KLK1*, *LTB4R*, *PSMB9*, *NAALAD2*), which achieved an average AUC of 0.999 in the training set and demonstrated robust diagnostic efficacy in three independent validation cohorts (GSE17818, GSE17895, GSE66272) with AUC values of 1.000, 0.967, and 0.985, respectively. Complementary performance metrics further confirmed the model’s utility (Supplementary Materials Table S4): Precision-Recall Area Under the Curve (PR AUC) values were 0.980 (95% CI: 0.947–0.991), 0.979 (95% CI: 0.953–0.992), and 0.871 (95% CI: 0.731–0.960) for GSE17818, GSE17895, and GSE66272, respectively, while Brier scores were 0.088 (95% CI: 0.061–0.115), 0.101 (95% CI: 0.074–0.127), and 0.043 (95% CI: 0.011–0.092). Calibration plots showed good agreement between predicted probabilities and observed outcomes across all cohorts (Figure 4H). Compared with baseline models, the glmBoost + RF model consistently outperformed a random classifier (expected AUC = 0.5), logistic regression without feature selection (AUC range: 0.80–0.89 across cohorts), and a model using only the top five DEGs (AUC range: 0.85–0.90), demonstrating its added predictive value (Supplementary Materials Table S5). To further explore the decision-making mechanism of the model and identify the most central driver genes, we conducted SHAP interpretability analysis to assess the role weights of each gene within this integrated model. The SHAP feature importance ranking indicated that *IGFBP3* (SHAP value = 0.117) and *ITGA5* (SHAP value = 0.078) were the two genes that contributed most significantly to the model’s predictions (Figure 4B). The SHAP summary plot illustrated the relationship between gene expression changes and the direction of model-predicted risk. Specifically, *IGFBP3*, *ITGA5*, *PYGL*, *LTB4R*, *MMP14*, and *PSMB9* were linked to an increased risk of ccRCC, suggesting that the expression of these genes drives the model to yield high-risk predictions. Conversely, elevated expression levels of *SLC22A8*, *KLK1*, and *NAALAD2* were associated with a reduced risk of ccRCC, suggesting their potential role as protective genes (Figure 4C). The SHAP-dependent scatter plots revealed that the contributions of genes such as *IGFBP3*, *ITGA5*, and *PYGL* to model predictions exhibited significant nonlinearity (Figure 4D). Notably, when the expression level of *ITGA5* exceeded 7, high expression of *IGFBP3* (greater than 10) corresponded to the highest SHAP contribution to the model (greater than 0.1). The SHAP value increased from −0.1 to 0.15 as the expression of both genes rose concurrently. A similar nonlinear relationship was observed between *PYGL* and *ITGA5*. Furthermore, as the expression of *SLC22A8* increased from 2 to 9, the level of *MMP14* decreased significantly (from approximately 9 to 3), with the corresponding scatter color transitioning from orange-red to dark purple. The SHAP value for this feature combination declined from 0.05 to −0.10, suggesting a synergistic negative regulatory effect of the two genes on model predictions. These observations provide insights into the specific mechanisms through which key genes and their expression patterns influence model predictions. Force-directed analysis indicated that *IGFBP3* (expression = 6.54, Δ = −0.151) and *ITGA5* (4.57, Δ = −0.083) were the primary contributors to the alteration in model predictions, serving as the principal negative regulators. Consequently, the model output (f(x) = 0.01) was significantly lower than the baseline prediction (E[f(x)] = 0.589) (Figure 4E). Based on the ranking of SHAP values, we selected the top five genes (*IGFBP3*, *ITGA5*, *PYGL*, *SLC22A8*, *LTB4R*) from the nine key genes as core genes for subsequent validation. The diagnostic ROC curves for these core genes demonstrated that their AUC values were *IGFBP3* (AUC = 0.982), *ITGA5* (AUC = 0.976), *PYGL* (AUC = 0.975), *SLC22A8* (AUC = 0.966), and *LTB4R* (AUC = 0.942). The optimal diagnostic threshold for each core gene was determined using the Youden index (sensitivity + specificity − 1), with corresponding sensitivity and specificity values reported in Supplementary Materials Table S6. All core genes demonstrated high sensitivity and specificity at their optimal thresholds, consistent with their strong diagnostic performance as indicated by AUC values. These findings suggest that the core genes effectively differentiate ccRCC tissues from normal tissues, exhibiting high independent diagnostic efficacy—by which we refer to their ability to distinguish ccRCC from normal tissues in datasets that were completely held out during model development (GSE17818, GSE17895, and GSE66272), which were never used for feature selection, model training, or hyperparameter tuning, ensuring that the reported performance reflects true generalizability rather than overfitting to the training data (Figure 4F). Furthermore, volcano plots of DEGs revealed that, under the threshold for differential expression screening (FDR-corrected *p* < 0.05 and |log_2_(FC)| > 0.585), the expression of *IGFBP3*, *ITGA5*, *PYGL*, and *LTB4R* was significantly up-regulated, while *SLC22A8* was significantly down-regulated (Figure 4G). These findings suggest that all core genes exhibited significant expression differences in ccRCC tissues.

### 2.5. Single-Cell Transcriptome Analysis of Core Genes

**Figure 5:**
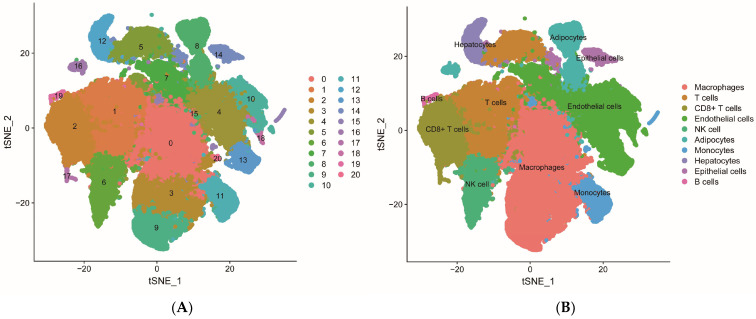

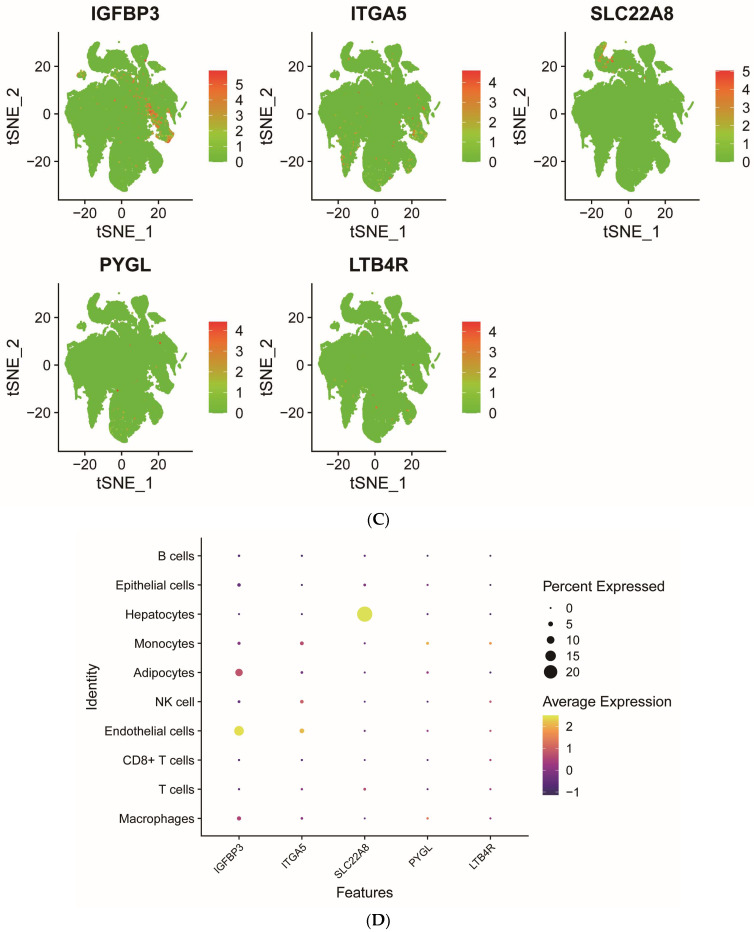
Results of single-cell transcriptome analysis of core genes. (**A**) The t-distributed stochastic neighbor embedding (t-SNE) downscaling plot presents a 2D visualization of high-dimensional single-cell data processed by the t-SNE algorithm. Each point corresponds to an individual cell, while clusters represented by differently colored numbers (0–20) indicate distinct cell groups identified by the clustering algorithm. (**B**) The t-SNE downscaling plot annotated by cell type illustrates the t-SNE visualization following cell type annotation for each cluster, based on cellular gene expression features, thereby visualizing the cellular composition of the sample through color-coded cell types. (**C**) The expression heatmap of core genes sequentially displays the expression levels of five core genes within the t-SNE downscaling space. Green indicates low expression, red signifies high expression, and the expression level of each gene is represented by a color gradient. (**D**) Bubble plots depict the expression patterns of the five core genes across various cell types. The *x*-axis denotes the gene name, the *y*-axis indicates the cell type, and the size of the dots reflects the proportion of gene expression in that cell type (larger dots correspond to higher proportions). The dot color represents the average expression intensity of the gene in that cell type, ranging from −1 (purple, low expression) to 2 (yellow, high expression). The “hepatocyte” cluster is likely a technical artifact representing metabolically active proximal tubular cells (see Section 2.5 for details).

The single-cell RNA sequencing (scRNA-seq) data from kidney tissue samples underwent rigorous quality control to eliminate low-quality cells and technical artifacts. In this study, the top 1500 genes exhibiting high coefficients of variation were selected for further analysis. Data dimensionality reduction was achieved through t-distributed stochastic neighbor embedding (t-SNE), which was combined with the Louvain clustering algorithm to categorize the cells. Cell types were annotated based on established marker genes, resulting in the identification of ten distinct cell clusters: macrophages, T cells, CD8+ T cells, endothelial cells, natural killer cells, adipocytes, monocytes, hepatocytes, epithelial cells, and B cells (Figure 5A,B). The cluster annotated as “hepatocytes” by automated reference-based annotation likely represents a metabolically active subpopulation of proximal tubular epithelial cells, as these cells co-expressed proximal tubular markers (e.g., *SLC22A6*, *SLC22A8*) and hepatocyte-associated metabolic enzymes (e.g., *ALDOB*, *FBP1*), leading to misclassification. The expression profiles of five core genes (*IGFBP3*, *ITGA5*, *PYGL*, *SLC22A8*, *LTB4R*) were further analyzed to explore their specific expression patterns across various cell types in ccRCC tissues. Quantitative analysis (Supplementary Materials Table S7) revealed that *IGFBP3* was expressed in 8.9% ± 9.6% of endothelial cells (mean expression among expressing cells: 2.76 ± 1.09) and *ITGA5* in 5.3% ± 3.6% of endothelial cells (mean expression: 2.28 ± 0.83), with both genes showing less than 2% expression in most other cell types, suggesting their potential roles in ccRCC angiogenesis and the regulation of the tumor microenvironment. *SLC22A8* exhibited higher expression levels in hepatocytes, with 18.8% ± 15.8% of hepatocytes expressing this gene (mean expression: 1.90 ± 1.18), compared to less than 2% in other cell types, suggesting possible ectopic expression during tumor progression or metastasis. Conversely, *PYGL* and *LTB4R* were predominantly identified in immune cells, including monocytes and macrophages, with *PYGL* expressed in 2.1% ± 2.3% of monocytes (mean expression: 1.37 ± 1.13) and *LTB4R* in 1.7% ± 2.1% of monocytes (mean expression: 1.31 ± 1.14), suggesting their potential roles in mediating tumor-associated inflammatory responses and immune regulation (Figure 5C,D). Collectively, these findings suggest that OTA exposure is associated with tumor cell metabolism, angiogenesis, immune regulation, and the inflammatory microenvironment, potentially through selective modulation of specific cell populations, which may contribute to the progression of ccRCC.

### 2.6. Molecular Docking Validation

**Figure 6:**
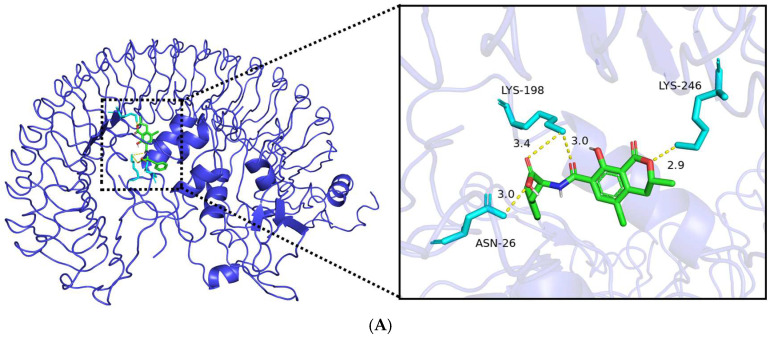

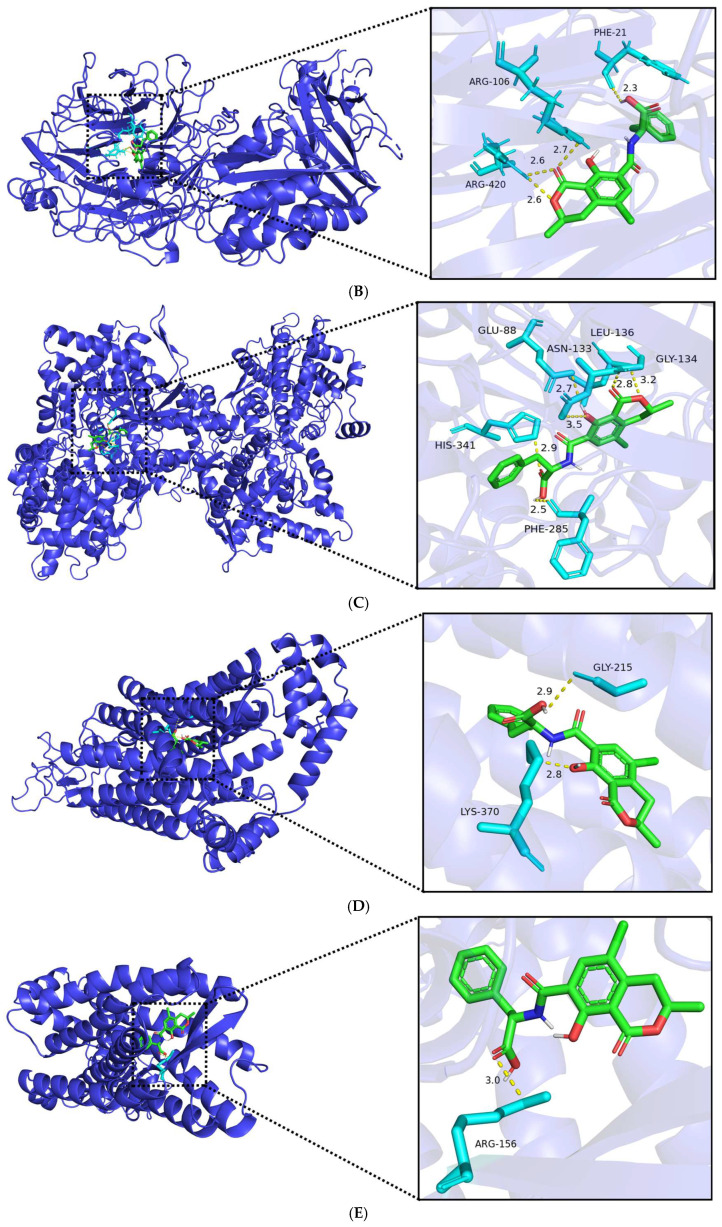
Molecular docking results of OTA with core target proteins: (**A**) OTA–IGFBP3; (**B**) OTA–ITGA5; (**C**) OTA–PYGL; (**D**) OTA–SLC22A8; (**E**) OTA–LTB4R. The molecular docking diagrams illustrate the three-dimensional structure of the high-affinity binding between OTA and the core protein targets. The global structure of each protein is depicted on the left side in dark blue, while the right side features local zoomed-in views that emphasize the key interaction regions between the ligand and the protein. The ligand is indicated in green, amino acid residues are shown in light blue, and their corresponding names are labeled with letters. The yellow dotted lines represent hydrogen bonding interactions, with the numbers adjacent to these lines denoting the hydrogen bonding distances.

To validate the reliability of our docking protocol, we performed redocking of native ligands for proteins with available co-crystallized structures. For PYGL, redocking of the native ligand (glucose) into the original structure (PDB 3CEJ) yielded an root-mean-square deviation (RMSD) of 0.62 Å, well below the acceptable threshold of 2.0 Å. For LTB4R, redocking of the co-crystallized antagonist (PDB 7K15) produced an RMSD of 1.13 Å. Redocking validation was limited to PYGL and LTB4R, as these were the only core targets with available high-resolution crystal structures containing co-crystallized native ligands in the PDB database; for IGFBP3 and ITGA5, the available structures (7WRQ and 4WJK) do not contain co-crystallized ligands suitable for redocking, and for SLC22A8, no experimental structure is available (AlphaFold model used). These results indicate that our docking parameters reliably reproduce experimentally observed binding modes. The interaction of OTA with the protein structures of core targets (IGFBP3, ITGA5, PYGL, SLC22A8, and LTB4R) was assessed through molecular docking. The results predicted that OTA demonstrated a strong binding affinity for all five target proteins, with binding energies all less than −5.0 kcal/mol (Table 1). It is essential to emphasize that these binding energy values are primarily estimates derived from the docking scoring function, rather than thermodynamic binding free energies obtained through experimental methods. Given that the molecular docking algorithm utilizes a simplified scoring function, it may not fully capture the complexity of protein–ligand interactions in biological systems. Furthermore, the visualization of binding conformations (Figure 6A–E) shows that all OTA–protein complexes maintain stable docking conformations, providing a basis for understanding potential intermolecular interactions.

## 3. Discussion

In recent years, the relationship between environmental toxins and cancer development has garnered significant attention. Among these toxins, OTA, a mycotoxin commonly found in food, may be closely linked to an increased risk of RCC, particularly ccRCC [21,22]. Existing studies indicate that the mechanisms underlying the carcinogenicity of OTA are complex and involve the synergistic effects of multiple pathways. Specifically, OTA can induce oxidative stress and DNA damage upon renal accumulation, which is considered a key mechanism of its nephrotoxicity [23]. OTA activates mitogen-activated protein kinases (MAPK), including apoptosis signal-regulated kinase 1 (ASK1), extracellular signal-regulated kinase 1/2 (ERK1/2), and c-Jun amino-terminal kinase (JNK); this activation contributes to the excessive generation of reactive oxygen species (ROS), disrupts cellular redox balance, and leads to DNA strand breaks in renal cells, thereby exacerbating oxidative stress and DNA damage [24,25]. Furthermore, OTA inhibits the activity of DNA repair enzymes such as pre-mRNA processing factor 18 (PRPF18), resulting in an impaired ability to effectively repair damaged DNA and further increasing the risk of gene mutations [24]. These findings suggest that the nephrotoxic and carcinogenic effects of OTA may promote tumor progression through mutations induced by oxidative DNA damage. In addition, OTA influences ERK1/2 phosphorylation and apoptosis-related proteins, such as caspase-3/7, by modulating critical signaling pathways, including phosphatidylinositol 3-kinase/protein kinase B (PI3K/Akt) and nuclear factor κB (NF-κB), and this modulation disrupts the equilibrium between cell proliferation and apoptosis, potentially contributing to the malignant transformation and oncogenic effects in renal epithelial cells [26,27]. Additionally, OTA promotes tumor progression by interfering with epigenetic regulatory mechanisms, such as DNA methylation and translational modifications, which result in altered gene function and malignant cell transformation [28]. Based on the findings of this study, the intersection of potential targets of OTA with ccRCC-related genes showed significant enrichment in pathways such as Rap1, TNF, and IgSF CAM. These pathways are intricately linked to tumor cell migration and invasion. Notably, IgSF CAM serves as a substrate to activate Rap1, which, upon activation, regulates the cell cycle and development by inhibiting the activity of the MAPK and PI3K pathways, thereby influencing the proliferation, migration, and invasive capabilities of tumor cells [29]. Furthermore, the activation of the TNF pathway can further modulate NF-κB expression, which subsequently impacts the balance of cell proliferation and apoptosis and is associated with tumor immune escape [29,30]. These observations suggest that OTA may facilitate the development of ccRCC through the synergistic effects of the aforementioned pathway network. In conclusion, OTA-associated ccRCC represents a complex process characterized by multiple targets and pathways, with extensive interactions among the underlying mechanisms. Consequently, further investigation into the key target genes and regulatory networks will enhance our understanding of the toxicological mechanisms of OTA and aid in the development of targeted prevention and treatment strategies for ccRCC.

In this study, we combined bioinformatics analyses of public transcriptomic data with in silico target prediction and molecular docking to systematically investigate the potential molecular mechanisms underlying OTA-associated ccRCC, utilizing bioinformatics techniques such as network toxicology, machine learning, and molecular docking. Initially, we screened potential targets from various public databases and identified a total of 56 intersecting target genes associated with OTA and ccRCC. Through GO and KEGG pathway enrichment analyses, we observed that OTA may facilitate the progression of ccRCC by modulating critical processes, including extracellular matrix remodeling, tumor immune microenvironment dynamics, signaling pathway transduction, as well as cellular metabolism and protein homeostasis. Subsequently, we constructed 113 predictive models employing machine learning methods and selected the “glmBoost + RF” integrated model, which exhibited the best overall performance (AUC > 0.99 in the training set). From this model, we extracted nine key ccRCC-related genes: *IGFBP3*, *ITGA5*, *SLC22A8*, *PYGL*, *MMP14*, *KLK1*, *LTB4R*, *PSMB9*, and *NAALAD2*. The rationality and clinical applicability of the machine learning model were further validated using the SHAP algorithm. The SHAP interpretability analysis indicated that *IGFBP3* (SHAP value = 0.117) and *ITGA5* (SHAP value = 0.078) were the most significant contributors to model predictions, suggesting that they may serve as core driver genes in ccRCC. These findings provided insights into the relationship between the expression levels of each gene and the risk of ccRCC, as well as the nonlinear contributions of these genes to the model output. This analysis revealed interactions and synergistic effects among the genes, offering potential mechanistic support for clinical translational research. Consequently, we selected five target genes (*IGFBP3*, *ITGA5*, *PYGL*, *SLC22A8*, and *LTB4R*) with the highest SHAP values as the core genes for the OTA–ccRCC association mechanism for further analysis. Diagnostic ROC curve analysis indicated that the AUC values for these five core genes ranged from 0.942 to 0.982, suggesting high independent diagnostic efficacy. Gene differential expression analysis revealed that the expression levels of *IGFBP3*, *ITGA5*, *PYGL*, and *LTB4R* were significantly upregulated in ccRCC tissues, while *SLC22A8* expression was significantly downregulated. These results aligned with the SHAP analysis and further supported the association of these core genes with ccRCC. In addition, single-cell transcriptome analysis further revealed the specific expression of core target genes across various cell types, suggesting their potential involvement in tumor cell metabolism, angiogenesis, immune regulation, and the inflammatory microenvironment. Additionally, molecular docking results predicted strong binding affinity between OTA and the five core targets, with binding energies all measuring less than −5.0 kcal/mol, suggesting a potential oncogenic role of OTA in ccRCC. The cell-type-specific expression patterns observed in our single-cell analysis align with the known biology of ccRCC and its putative cell of origin. ccRCC is thought to arise from proximal tubular epithelial cells, yet our analysis revealed that the core genes most strongly associated with OTA exposure—particularly *IGFBP3* and *ITGA5*—were predominantly expressed in endothelial cells and immune cells rather than in epithelial cells (which encompass renal tubular populations) (Supplementary Materials Table S7). This finding suggests that OTA may exert its oncogenic effects not directly on the cell of origin, but through modulation of the tumor microenvironment, including angiogenesis (via endothelial cells) and immune regulation (via macrophages and monocytes). This interpretation is consistent with recent studies highlighting the critical role of the tumor microenvironment in ccRCC progression. For epithelial cells representing the renal tubular compartment—the primary site of OTA accumulation and toxicity—we observed moderate expression of *PYGL* and *SLC22A8*, the latter being a known organic anion transporter involved in xenobiotic clearance. The downregulation of *SLC22A8* in ccRCC tissues (Figure 4G) and its expression in epithelial cells (Figure 5D) support its potential role as a detoxification-related gene whose loss may contribute to OTA-associated carcinogenesis. Notably, a subset of these epithelial cells exhibited high metabolic activity and were misclassified as “hepatocytes” by automated annotation (see Section 2.5), further underscoring the metabolic heterogeneity within the renal tubular compartment. These spatially resolved expression patterns provide mechanistic insights into how OTA exposure may promote ccRCC through both direct tubular effects and microenvironmental remodeling. Among the pathways uniquely enriched in the OTA–ccRCC overlap, Rap1 signaling and IgSF CAM interactions stood out as particularly relevant to ccRCC pathogenesis. Rap1 is a key regulator of cell adhesion and migration, and its activation has been implicated in renal cancer progression. IgSF CAMs mediate cell–cell interactions and can promote epithelial–mesenchymal transition (EMT) when dysregulated. The specific enrichment of these pathways in the OTA–ccRCC overlap—but not in ccRCC DEGs alone or OTA targets alone—suggests that OTA exposure may uniquely potentiate these oncogenic mechanisms, distinguishing its effect from general ccRCC biology. In conclusion, the five core genes identified in this study may serve as key molecular targets for OTA-associated ccRCC development. It is important to note that, as an in silico exploratory study, our findings should be interpreted as hypothesis-generating rather than definitive evidence of causality. Nonetheless, these results provide a foundation for future experimental validation and mechanistic studies.

The core genes identified in this study demonstrated diverse roles in OTA-associated ccRCC. Insulin-like growth factor binding protein 3 (IGFBP3) is a multifunctional secreted glycoprotein that can form a complex with IGF-1/IGF-2, thereby regulating cell growth, survival, and metabolism, while also exerting pro-apoptotic or pro-tumorigenic effects depending on specific tissues and microenvironments through direct interactions with membrane receptors or intranuclear targets [31]. Liu et al. [32] discovered that targeted inhibition of IGFBP3 expression in human ccRCC xenograft mice disrupted the IGFBP3-AKT/signal transducer and activator of transcription 3 (STAT3)/MAPK-Snail axis, leading to significant improvements in ccRCC cell proliferation, migration, and epithelial–mesenchymal transition (EMT). This finding confirms that IGFBP3 acts as a pro-tumorigenic cytokine in ccRCC. In this study, *IGFBP3* emerged as the predictor with the highest SHAP value, with its elevated expression significantly correlating with an increased risk of ccRCC. ROC analysis demonstrated its robust diagnostic efficacy, while single-cell transcriptome analysis suggested its involvement in tumor microenvironmental regulation. Additionally, molecular docking predicted that OTA could stably bind to IGFBP3, findings that align closely with previous research. Consequently, we hypothesize that in OTA-associated ccRCC, IGFBP3 may serve as a crucial link between toxic stress and pro-proliferative as well as pro-invasive signals. We propose that OTA may enhance pro-cancer signals by upregulating IGFBP3 expression or directly activating its downstream pathways. Furthermore, the high expression of IGFBP3 is expected to facilitate the expansion and metastasis of malignant clones, functioning as a “molecular amplifier” in the transition from nephrotoxicity to nephrocarcinogenicity induced by OTA.

Integrin subunit alpha 5 (ITGA5), a member of the integrin family, functions by forming a heterodimer (α5β1) with the β1 subunit [33]. As the primary receptor for fibronectin, it mediates cell adhesion to the ECM, participates in ECM remodeling, facilitates cell migration and invasion, and activates signaling pathways such as focal adhesion kinase/ proto-oncogene tyrosine-protein kinase Src (FAK/Src) and PI3K/AKT, thus serving as a crucial molecule in promoting the progression of various tumors [34]. Che et al. [35] demonstrated through in vitro experiments that ITGA5 is significantly upregulated in ccRCC, with its high expression correlating with ccRCC progression and poor prognosis. Conversely, downregulation of ITGA5 expression inhibited ccRCC cell proliferation, migration, and angiogenesis, while also altering immune cell infiltration patterns. This suggests that ITGA5 may serve as a potential therapeutic target for ccRCC. In this study, GO analysis revealed significant enrichment in the ECM remodeling process, and single-cell transcriptome analysis indicated that *ITGA5* could play a role in tumor angiogenesis and regulation of the tumor microenvironment. Furthermore, *ITGA5* is identified as a core gene with a high SHAP value, and its elevated expression aligns with an increased risk of ccRCC. Molecular docking studies predicted that OTA can bind stably to ITGA5, corroborating previous findings. It is hypothesized that ITGA5 may facilitate the transformation of renal tubular epithelial cells into an invasive phenotype by enhancing the adhesion and remodeling of tumor cells within the ECM. Meanwhile, ITGA5 not only engages with immune-related pathways via integrin downstream signaling to remodel the immune microenvironment and promote immune evasion, but also stimulates the expression of angiogenesis-related molecules, such as vascular endothelial growth factor (VEGF). This dual action may synergistically regulate tumor vascularization and microenvironmental homeostasis, suggesting a potential molecular mechanism of “OTA → ECM/immune microenvironment/angiogenesis → ccRCC progression.” This understanding offers a potential target for subsequent targeted interventions.

Glycogen phosphorylase L (PYGL), a rate-limiting enzyme in glycogenolysis, serves as a critical molecular node for maintaining glucose homeostasis and cellular energy supply within the organism. It aids cells in responding to hypoxia and nutrient deprivation and is recognized as a key molecule in the metabolic reprogramming of tumors [36]. Li et al. [37] reported a significant upregulation of PYGL expression in human ccRCC samples. They demonstrated that silencing PYGL expression inhibited ccRCC cell proliferation and migration while reducing tumor resistance to targeted therapies. This suggests that PYGL plays a vital role in tumor progression and the development of drug resistance, positioning it as a potential therapeutic target for ccRCC. In conjunction with the findings of this study, high *PYGL* expression correlates with an increased risk of ccRCC and serves as an important predictive gene with a high SHAP value. Additionally, single-cell transcriptome analysis indicates its involvement in remodeling the immune microenvironment, while molecular docking studies suggest that OTA can bind stably to PYGL. Based on this, we hypothesized that following OTA-induced stress in renal tubular epithelial cells, these cells enhance glycogenolysis and glycolysis by upregulating PYGL expression to supply adequate substrates for biomolecule synthesis. This metabolic reprogramming may not only aid damaged cells in evading apoptosis but also promotes their transformation into a malignant phenotype. Consequently, PYGL serves as a crucial link in the mechanisms underlying ccRCC development and drug resistance associated with OTA exposure through metabolic reprogramming. These findings offer a theoretical foundation and potential intervention target for future studies.

Solute carrier family 22 member 8 (SLC22A8), also known as organic anion transporter 3 (OAT3), belongs to the solute carrier family 22 and is prominently expressed in the basolateral membrane of the proximal renal tubule. It plays a crucial role in clearing various endogenous and exogenous organic anions, participating in the transportation of uremic toxins and solutes, thus serving as a pivotal detoxification transporter protein [38,39]. Chen et al. [40] illustrated that SLC22A8 expression was notably reduced in ccRCC patients compared to the control group. The diminished expression was linked to shorter overall survival and unfavorable prognosis, indicating its potential as a protective gene in ccRCC. Consistent with previous prognostic analyses, our study revealed a significant down-regulation of *SLC22A8* in ccRCC tissues, where its elevated expression correlated with a lower disease risk. Furthermore, ROC analysis underscored the robust diagnostic capability of SLC22A8, while molecular docking simulations predicted a stable binding of OTA to this transporter. The decline in SLC22A8 expression is speculated to serve as a reverse indicator of compromised renal tubular differentiation and detoxification, suggesting a potential pathological mechanism of renal dysfunction induced by OTA exposure, subsequently propelling ccRCC progression at the molecular level. These findings suggest that SLC22A8 holds promise as a potential biomarker for OTA-related ccRCC.

Leukotriene B4 receptor 1 (LTB4R), a high-affinity leukotriene B4 receptor (BLT1), is implicated in the regulation of inflammatory responses and immune infiltration through LTB4R signaling, and contributes to the establishment of an inflammatory oncogenic microenvironment across various tumors [41]. Wu et al. [42] reported that LTB4R expression was significantly elevated in ccRCC tissues compared to normal renal tissues, with high expression correlating with advanced tumor stage and poor prognosis. This expression pattern may enhance tumor cell proliferation and inhibit apoptosis, suggesting that LTB4R could serve as a novel immune-related biomarker and a potential therapeutic target for ccRCC. In alignment with the current study, we observed that *LTB4R* exhibited a high SHAP predictive value and independent diagnostic value (AUC = 0.942), with elevated expression linked to an increased risk of ccRCC. Single-cell transcriptome analysis suggested its involvement in tumor-associated inflammatory responses and the remodeling of the immune microenvironment. Additionally, molecular docking results predicted stable binding between OTA and LTB4R. We hypothesize that OTA exposure may induce chronic inflammation and immune dysregulation in the kidney, subsequently upregulating LTB4R expression. This upregulation could amplify LTB4R-mediated pro-inflammatory and pro-proliferative signals, drive immune cells toward a suppressive phenotype, and ultimately accelerate the progression of ccRCC. These observations suggest that LTB4R may serve as a critical link between “toxic inflammation” and “tumor signaling” in OTA-associated ccRCC. Future in vitro and in vivo experiments could further elucidate its mechanism of action.

**Figure 7:**
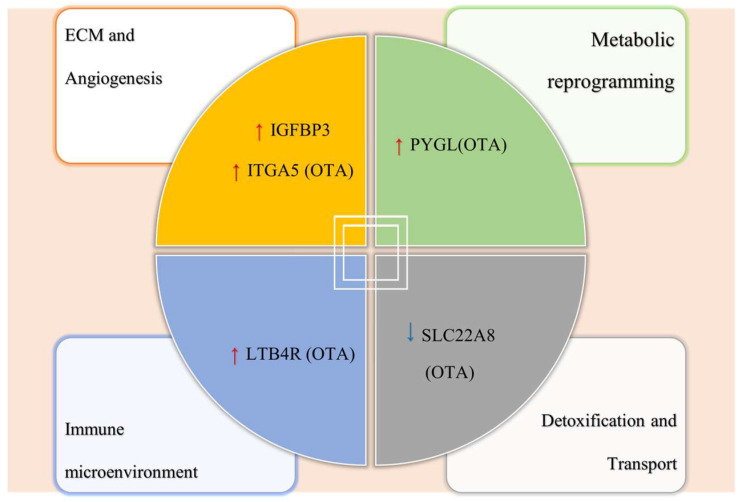
Schematic summary of core gene functions in ccRCC pathways. This diagram establishes a conceptual framework for elucidating the potential roles of OTA–core gene interactions in ccRCC progression, where the five core genes (*IGFBP3*, *ITGA5*, *PYGL*, *SLC22A8*, *LTB4R*) are mapped to four key biological processes underlying clear cell renal cell carcinoma (ccRCC) pathogenesis—extracellular matrix (ECM) remodeling and angiogenesis (orange), metabolic reprogramming (green), immune microenvironment modulation (blue), and detoxification/transport (gray)—with red upward arrows denoting up-regulation in ccRCC tissues, blue downward arrows denoting down-regulation, and “OTA” labels indicating molecular docking-predicted stable binding of OTA to all five proteins.

To provide a visual integration of how these five core genes may collectively contribute to ccRCC pathogenesis, we constructed a schematic diagram summarizing their involvement in key biological processes relevant to renal cancer biology (Figure 7). The figure organizes the core genes into four major modules: ECM remodeling and angiogenesis (*ITGA5*, *IGFBP3*), metabolic reprogramming (*PYGL*), immune microenvironment modulation (*LTB4R*), and detoxification/transport (*SLC22A8*). It also indicates the direction of gene expression changes observed in ccRCC tissues (red upward arrows: up-regulated; green downward arrows: down-regulated) and the predicted binding of OTA to each protein based on molecular docking. This conceptual framework is intended to guide future experimental investigations into the potential mechanistic links between OTA exposure and ccRCC progression.

OTA is recognized as a nephrotoxin that frequently disrupts essential cellular functions, including protein synthesis through the inhibition of phenylalanine-tRNA synthetase and mitochondrial respiration. Our molecular docking analysis indicated stable binding of OTA to all five core proteins, prompting an inquiry into whether these interactions are inhibitory and how such inhibition may facilitate, rather than hinder, the progression of ccRCC. We propose a dual-mechanism hypothesis to reconcile this apparent contradiction. First, for genes that are upregulated in ccRCC and possess pro-tumorigenic functions (*IGFBP3*, *ITGA5*, *PYGL*, *LTB4R*), OTA binding may not merely inhibit their activity but could also disrupt their normal regulatory networks. For instance, *IGFBP3* can have either pro- or anti-tumorigenic effects depending on post-translational modifications and binding partners; OTA interference might shift the balance toward a pro-carcinogenic signaling mode. Similarly, the interaction of *ITGA5* with OTA may alter integrin conformation and downstream FAK/PI3K signaling in ways that paradoxically enhance cell migration and invasion. Second, for the protective gene *SLC22A8*, which is downregulated in ccRCC and encodes a critical organic anion transporter responsible for renal toxin clearance, OTA binding is likely to inhibit its transport activity. This inhibition would further compromise the kidney’s detoxification capacity, leading to the accumulation of OTA and other nephrotoxic compounds, thereby fostering a microenvironment conducive to DNA damage, oxidative stress, and malignant transformation. The combined effect of disrupting tumor-suppressive regulation in protective genes and aberrantly modulating pro-tumorigenic genes may synergistically enhance the initiation and progression of ccRCC. We emphasize that this hypothesis is grounded in computational predictions and necessitates experimental validation; however, it offers a conceptual framework for understanding how the predominantly inhibitory interactions of OTA could ultimately lead to oncogenic outcomes.

The synergistic effects of the aforementioned core target genes suggest a multidimensional complex network that encompasses cell growth regulatory signaling, ECM remodeling, immune evasion, metabolic reprogramming, chronic inflammation, and detoxification functions. This network offers a potential mechanistic framework for elucidating the nephrotoxicity and oncogenicity of OTA in ccRCC, while also providing insights and references for subsequent targeted interventions.

In this study, we systematically investigated the potential targets and mechanisms of action of OTA-associated ccRCC using network toxicology and machine learning techniques. We developed a multilevel closed-loop research framework that significantly enhanced the reliability of the predictive outcomes and offered a novel perspective on the relationship between OTA and ccRCC. Nonetheless, the study has several limitations: (1) Although the machine learning prediction model exhibits a high confidence level, its biological relevance requires further validation through in vivo and in vitro experiments to establish its reliability in actual biological systems. (2) The network toxicology analysis primarily relies on static data and does not account for the temporal aspects of OTA exposure or the dose–response relationship, necessitating further evaluation through the integration of dynamic data. It is important to emphasize that molecular docking provides computational predictions of binding affinity and does not establish binding under physiological conditions. The docking scores reported are estimates derived from a simplified scoring function and may not fully capture the complexity of protein–ligand interactions in biological systems. Therefore, these results should be interpreted as suggestive and hypothesis-generating, providing a basis for future experimental validation. (3) The molecular docking analysis employed a semi-flexible docking strategy, permitting only the ligand to be flexibly adjusted while neglecting the complete flexibility of the receptor, particularly the side chains of the binding site. Furthermore, only the top-ranked docking conformations were analyzed, potentially overlooking superior binding modes and dynamic interaction characteristics. (4) This study lacked external validation based on real-world clinical cohort data, relying instead on independent validation from other cohorts within the same database, which may compromise the reliability of the model’s predictive results.

Given the aforementioned limitations, future research should leverage advanced technologies, such as single-cell sequencing, gene knockdown, and organoids, while conducting in vivo and ex vivo functional experiments in conjunction with multi-omics analysis. This approach aims to further explore the specific molecular mechanisms underlying the core genes involved in the development of ccRCC induced by OTA. Additionally, experiments employing multi-dose gradient exposure and time series designs can be implemented to quantitatively evaluate the dynamic expression of core genes, the activation of related pathways, and alterations in disease phenotypes, thereby revealing their temporal and quantitative effects. Furthermore, flexible docking, multi-configuration analysis, and molecular dynamics simulations can be integrated to account for the flexibility of receptor binding site side chains, systematically exploring various ranked docking conformations to comprehensively analyze the dynamic characteristics and conformational diversity of protein interactions. Furthermore, decision curve analysis should be incorporated in subsequent clinical studies to evaluate the net benefit of the core gene panel and assess its potential utility in guiding clinical decision-making. Finally, large-scale, multicenter, standardized clinical cohort studies should be conducted to further validate the external efficacy and clinical applicability of the prediction model using multicenter data, thereby enhancing the feasibility of clinical translation. Overall, this study establishes a theoretical foundation for elucidating the potential mechanisms of OTA-associated ccRCC, and the findings can be substantiated through comprehensive functional experiments and clinical studies in the future, ultimately supporting the toxicity assessment of OTA and the development of prevention and treatment strategies for ccRCC.

## 4. Materials and Methods

### 4.1. OTA Chemical Composition and Target Collection

The keyword “Ochratoxin A” was searched in the PubChem database (https://pubchem.ncbi.nlm.nih.gov/; last accessed: 9 December 2025) to obtain its chemical structure and SMILES formula (C[C@@H]1CC2=C(C=C(C(=C2C(=O)O1)O)C(=O)N[C@@H](CC3=CC=CC=C3)C(=O)O)Cl). Utilizing this molecular information, the analysis of potential targets was conducted using the ChEMBL (https://www.ebi.ac.uk/chembl/; last accessed: 9 December 2025), SwissTargetPrediction (http://www.swisstargetprediction.ch/; last accessed: 9 December 2025), and SEA (https://sea.bkslab.org/; last accessed: 9 December 2025) databases. For ChEMBL, all human target genes with reported bioactivity were retained. For SwissTargetPrediction, targets with a probability score > 0.05 were included; this threshold balances sensitivity and specificity by retaining high-confidence predictions while excluding low-probability hits. For SEA, targets with a maximum Tanimoto coefficient (max tc) > 0.05 were retained, consistent with the database’s default significance threshold. Subsequently, the UniProt database (https://www.uniprot.org/uniprotkb; last accessed: 9 December 2025) was employed to convert all target identifiers to official HUGO Gene Nomenclature Committee (HGNC) symbols using the UniProt ID mapping tool, thereby resolving gene symbol ambiguities and ensuring standardized nomenclature. Genes with multiple entries arising from different database identifiers or synonymous symbols were consolidated, resulting in a consolidated target gene set of 232 unique OTA target genes.

To assess the specificity of OTA target prediction, we performed a negative control analysis using OTB, a structurally similar mycotoxin lacking the chlorine atom present in OTA and exhibiting substantially lower toxicity. The SMILES formula (C[C@@H]1CC2=C(C(=C(C=C2)C(=O)N[C@@H](CC3=CC=CC=C3)C(=O)O)O)C(=O)O1) for OTB was obtained from PubChem, and the same target prediction pipeline described was applied (ChEMBL, SwissTargetPrediction, SEA; probability/score > 0.05). The resulting OTB target genes were intersected with the ccRCC-associated genes. The overlap size and enriched pathways were compared with those obtained for OTA.

### 4.2. Collection and Processing of ccRCC Transcriptome Data in the GEO Database

We searched the GEO database (https://www.ncbi.nlm.nih.gov/geo/; last accessed: 11 December 2025) using the keyword “clear cell renal cell carcinoma”, with data type restricted to Series, experimental technique set to “Expression profiling by array”, and requiring sufficient cancer versus non-cancer adjacent tissue samples, and obtained five ccRCC transcriptomic datasets: GSE16449 (platform GPL6480, 18 normal and 52 tumor samples), GSE53757 (platform GPL570, 72 normal and 72 tumor samples), GSE17818 (platform GPL9101, 13 normal and 102 tumor samples), GSE17895 (platform GPL9101, 22 normal and 138 tumor samples), and GSE66272 (platform GPL570, 27 normal and 27 tumor samples). Based on sample size and platform heterogeneity, GSE16449 and GSE53757, which have larger sample sizes, were designated as training cohorts, while GSE17818, GSE17895, and GSE66272 served as independent validation cohorts and were processed individually without merging or batch correction to preserve their independence for subsequent model validation. Although the two training datasets were generated on different microarray platforms (GPL6480 and GPL570), they were integrated at the gene level by retaining only genes present in both platforms, a common strategy for cross-platform data integration [43]. Subsequent batch correction was applied as described below. Clinical covariates (e.g., age, sex, tumor stage) were not consistently available across datasets and therefore were not included in the analysis, which we acknowledge as a limitation. All parameter choices for downstream analyses, including those for differential expression and WGCNA, followed established methodologies in the field [44]. To ensure data consistency and reliability, probe IDs were initially mapped to standard gene symbols according to the official annotation files of each microarray platform. In cases where multiple probes corresponded to the same gene, the maximum expression values were retained to correct the individual microarray expression data. Subsequently, the “limma (version 3.66.0)” package in R 4.5.1 software (R Foundation for Statistical Computing, Vienna, Austria) was utilized to merge the two training datasets, retaining only the intersecting genes to construct the merged expression matrix. This matrix underwent quantile normalization and log_2_ transformation to eliminate technical noise and standardize the data distribution. Quantile normalization was applied to ensure identical expression distributions across samples, thereby removing technical variation while preserving biological differences. To eliminate batch effects between the two training datasets, we applied ComBat batch correction within an empirical Bayesian framework using the “sva” package (version 3.58.0). ComBat adjusts for known batch effects using empirical Bayes estimates, making it robust even with relatively small sample sizes. The effectiveness of this process was then verified by PCA. As shown in Figure 2A,B, prior to correction, samples clustered by dataset origin, indicating substantial batch effects. After correction, samples from both datasets intermingled while maintaining clear biological separation between tumor and normal tissues, confirming that technical variance was successfully controlled and that downstream analyses reflect genuine biological signals rather than platform artifacts. This processed expression matrix was deemed suitable for subsequent differential expression and WGCNA analyses.

### 4.3. Gene Differential Expression Analysis

The batch-corrected transcriptome data were analyzed for differential expression using the “limma (version 3.66.0)” package in R. In this study, ccRCC tissue samples were compared with normal tissue samples. The significance thresholds were established at FDR-corrected *p* < 0.05, corrected for FDR, and |log_2_(FC)| > 0.585, corresponding to a 1.5-fold difference in expression. These thresholds were chosen to balance statistical rigor (via FDR correction) with biological relevance (via a moderate fold-change cutoff), following standard practices in transcriptomic studies [45]. DEGs were identified using the “pheatmap (version 1.0.13)” and “ggplot2 (version 4.0.1)” packages in R. Subsequently, the heatmap and volcano plot of DEGs were generated using the same packages, which visualized the clustering characteristics of gene expression and highlighted the significance of differential expression among the samples.

### 4.4. Weighted Gene Co-Expression Network Analysis (WGCNA)

The gene co-expression network was constructed using the “WGCNA (version 1.73)” package in R. Hierarchical clustering (CutHeight = 20,000) was employed to eliminate outlier samples, thereby ensuring data quality. The soft-threshold weights (power values) were established using the dynamic tree-cutting method with the cutreeDynamic function, with the criterion being that the scale-free topology fit index (R2) exceeded 0.85 for the first time, ensuring that the network adhered to the scale-free property. The optimal soft-threshold power was automatically selected as β = 9 by the pickSoftThreshold function. Subsequently, based on the selected soft threshold, the neighbor-joining matrix and topological overlap matrix (TOM) for all genes were calculated, followed by hierarchical clustering of genes based on TOM dissimilarity. The dynamic tree cutting method, implemented through the cutreeDynamic function, was employed to identify co-expression modules. Parameters, including minModuleSize = 60 and mergeCutHeight = 0.25, were established to facilitate the visualization of the gene clustering tree and module assignment results using the plotDendroAndColors function. To assess the robustness of these parameter choices, we performed a sensitivity analysis by varying the soft-threshold power (β = 8–10) and module merging height (mergeCutHeight = 0.20–0.30) while applying the same gene-level filters (kME > 0.8, GS > 0.2). For each combination, we calculated the union of filtered module genes and DEGs and determined its overlap with the predicted OTA targets. The results of this sensitivity analysis are provided in Supplementary Materials Table S1. The first principal component of each module was extracted as the module characteristic gene (ME). Subsequently, the Pearson correlation coefficients between the ME of each module and the target phenotypes were calculated. Modules exhibiting significant correlations were identified (|R| > 0.5, *p* < 0.05) and visualized using the labeledHeatmap function, resulting in a module–trait association heatmap. Finally, core genes for each module were selected based on module membership (kME > 0.8) and gene significance (GS > 0.2). The module genes demonstrating the most significant correlation with phenotypes, characterized by the smallest *p*-values and |R| > 0.5, were designated as the candidate gene set for subsequent functional analysis. Following these selection criteria, genes from the blue module (the module most significantly correlated with ccRCC) were retained as WGCNA-derived module genes for subsequent analysis.

### 4.5. Integration of ccRCC Key Target Genes

The genes identified through the analyses of DEGs and WGCNA were combined to form the key target gene set for ccRCC. Specifically, we took the union of the DEGs identified in Section 4.3 and the module genes obtained from WGCNA (Section 4.4), removing duplicates to yield a final set of ccRCC-associated target genes. This union strategy captures a comprehensive set of candidate genes by integrating evidence from both differential expression and co-expression network analysis. To illustrate the overlapping and independent distribution characteristics of these two gene types, a Venn diagram was generated using the “ggvenn (version 0.1.19)” package in R.

### 4.6. OTA and ccRCC Intersection Target Gene Screening

The OTA target gene set identified in Section 4.1 was intersected with the key target gene set for ccRCC screened in Section 4.5. This process yielded the target genes of OTA that are implicated in ccRCC. The intersection results were visualized using Venn diagrams generated by the “ggvenn (version 0.1.19)” package in R. Additionally, we produced network relationship files for “compound (OTA)-intersecting target gene-disease (ccRCC)” and files containing the attributes of the three types of nodes for further analysis. The Venn diagram was drawn again using the “ggvenn (version 0.1.19)” package to illustrate the intersection results, and the corresponding network relationship and attribute files were prepared for subsequent analysis.

### 4.7. OTA–ccRCC Association Network Construction

The network relationship files and node attribute files generated in 4.6 were imported into Cytoscape version 3.10.3 (Cytoscape Consortium, La Jolla, CA, USA) and mapped based on three node attributes: “Disease (ccRCC),” “Compound (OTA),” and “Gene (intersecting target gene).” These attributes were classified and mapped, resulting in the construction and visualization of the OTA–ccRCC association network, which is connected by the intersecting target genes.

### 4.8. GO and KEGG Enrichment Analysis of Intersecting Target Genes

The “clusterProfiler (version 4.18.4)” package in R, in conjunction with the “Org.Hs.eg.db” human gene annotation database, was employed to conduct GO functional annotation and KEGG pathway enrichment analysis. For all enrichment analyses, the background gene set was defined as all protein-coding genes in the human genome. *p*-values were adjusted for multiple testing using the Benjamini–Hochberg (BH) method to control the FDR, and adjusted *p* < 0.05 was considered statistically significant. The analysis was first performed on the intersecting target genes of OTA and ccRCC. The GO annotations encompassed (BP, CC, and MF, which facilitated the elucidation of the biological processes, subcellular localization, and molecular functions associated with the target genes. Concurrently, the KEGG enrichment analysis aimed to identify the core metabolic pathways and signaling mechanisms pertinent to the target genes, thereby enabling a deeper understanding of the molecular pathways involved in the regulation of ccRCC by OTA. To assess the specificity of the enrichment patterns, KEGG enrichment analysis was also performed on ccRCC DEGs alone and OTA targets alone using the same parameters and statistical thresholds. The results were visualized using the “enrichplot (version 1.30.4)” package, which illustrated the enriched functional categories and pathways.

### 4.9. Machine Learning Model Construction and Core Gene Screening

In this study, a multi-algorithmic machine learning prediction framework was employed to identify core genes associated with OTA-related ccRCC. To ensure the reproducibility and stability of the findings, all machine learning models were trained and cross-validated using a fixed random seed (set.seed(123)) to control for randomness. To ensure rigorous evaluation and prevent information leakage, we strictly separated data used for model development from data used for validation. The training cohort (GSE16449 and GSE53757, combined and batch-corrected as described in Section 4.2) was used exclusively for feature selection, model training, and internal cross-validation. Three independent datasets—GSE17818, GSE17895, and GSE66272—were held out as external validation cohorts and were not accessed during any stage of feature selection or model training. These external datasets were processed individually without merging or batch correction to preserve their independence and to test the model’s generalizability across different cohorts and platforms. Initially, utilizing a pre-divided training set along with three independent external validation sets, a two-step modeling strategy comprising “feature selection + classifier” was implemented during the training phase. Specifically, various algorithms, including RF, Ridge, Lasso, Stepglm, glmBoost, Support Vector Machine (SVM), Enet, Gradient Boosting Machine (GBM), Linear Discriminant Analysis (LDA), XGBoost, and NaiveBoost, were first applied for feature selection, followed by the construction of the corresponding classification model based on the selected genes. The R packages used for these algorithms were: randomForest (version 4.7.1.2) for RF, glmnet (version 4.1.10) for Ridge/Lasso/Enet, mboost (version 2.9.11) for glmBoost, e1071 (version 1.7.17) for SVM and NaiveBayes, gbm (version 2.2.2) for GBM, MASS (version 7.3.65) for LDA, and xgboost (version 3.1.2.1) for XGBoost. Ultimately, a total of 113 model combinations were generated through the integration of different feature selection methods and classification algorithms. For each combination, feature selection and classifier training were performed sequentially: the feature selection algorithm identified a subset of genes, and the classifier was then trained on those selected features. This sequential process was conducted separately within each nested cross-validation fold, and the final model for each combination was locked before any evaluation on external validation sets. The term “algorithm X + algorithm Y” (e.g., “glmBoost + RF”) thus refers to this sequential combination of a feature selection algorithm followed by a classifier, not to an ensemble that combines predictions from multiple models. To prevent information leakage and ensure unbiased performance estimation, we performed parameter optimization strictly within the training set. For algorithms requiring hyperparameter tuning (e.g., Enet, Lasso, Ridge, GBM, glmBoost, LDA), we applied 10-fold cross-validation on the training data to select optimal parameters. For other algorithms, feature selection was conducted directly on the training set using algorithm-specific importance measures (e.g., Random Forest variable importance, XGBoost gain). After all training decisions were finalized, the locked model was evaluated once on each of the three independent external validation cohorts, which had never been used during model development. Subsequently, the performance of all models was assessed using three independent external validation sets. The AUC served as the primary evaluation metric. To provide a comprehensive assessment, we additionally calculated the area under the PR AUC and the Brier score as measures of discrimination and calibration, respectively. Uncertainty was quantified by reporting 95% confidence intervals for all metrics via 1000 bootstrap resamples. Model performance was compared against several baseline models to contextualize the results, including a random classifier (expected ROC AUC = 0.5), logistic regression without feature selection (represented by the Stepglm models), and a model using only the top five DEGs. Models were ranked based on the average AUC value, and the combination of models exhibiting the highest average AUC (AUC > 0.9) was identified as the optimal model. The feature genes selected by this model were extracted as candidate core genes. Finally, the “ComplexHeatmap (version 2.26.0)” package in R was employed to generate a heatmap illustrating the AUC performance of each model, thereby visualizing the performance distribution across both the training set and the external validation set.

### 4.10. Model Interpretability Analysis and Validation of Core Gene Diagnostic Efficacy

To address the inherent “black box” nature of machine learning models and enhance the interpretability of model predictions, this study employed the SHAP algorithm to quantify the contribution of each feature gene. Initially, SHAP values for each gene were calculated using the “kernelshap (version 0.9.1)” package in R, based on core gene expression data obtained from preliminary screening. This quantification assessed both the positive and negative contributions of the genes to model predictions, as well as their relative importance. Subsequently, the “shapviz (version 0.10.3)” and “ggplot2 (version 4.0.1)” packages facilitated visualization and analysis, enabling the creation of SHAP feature importance histograms, summary plots, dependency scatter plots, and force-directed plots to elucidate the prediction results for individual samples. Core genes with higher contributions were ranked and selected for further analysis based on their average absolute SHAP values. To further validate the diagnostic potential of the core genes in ccRCC, volcano plots were generated based on transcriptome-wide differential analysis results, utilizing the “ggplot2 (version 4.0.1)” package in R. These plots emphasized the differential expression patterns of the core genes. Subsequently, the diagnostic efficacy of the core genes was assessed by employing normalized individual gene expression values as predictor variables, while the group classifications of ccRCC patients and normal controls served as outcome variables. ROC curves were constructed using the “pROC (version 1.19.0.1)” package in R. The optimal diagnostic threshold for each core gene was determined using the Youden index (sensitivity + specificity − 1), implemented via the coords function in the pROC package. The corresponding sensitivity and specificity values at these optimal thresholds were extracted and reported in Supplementary Materials Table S6. An AUC greater than 0.80 was considered indicative of significant diagnostic value for the core genes.

### 4.11. Single-Cell Transcriptome Analysis of ccRCC

To elucidate the expression patterns of core target genes at the single-cell level, this study utilized the single-cell dataset GSE242299 from the GEO database, analyzing 50,236 cells derived from 17 individuals through single-cell RNA sequencing (scRNA-seq). This dataset includes both ccRCC tumor samples and adjacent normal kidney tissues, comprising 8 tumor samples and 9 normal samples from 17 donors. Clinical information such as tumor grade and stage was not consistently available for all samples and therefore could not be included in the analysis. Initially, the raw data underwent quality control using the “Seurat (version 5.4.0)” package in R to exclude cells with fewer than 100 detected genes or more than 10% mitochondrial gene expression, thereby ensuring data reliability. Data were normalized using the LogNormalize method with a scale factor of 10,000. To identify the most biologically variable genes, we selected the top 1500 genes exhibiting high coefficients of variation. Subsequently, the data were standardized and normalized using “Harmony (version 1.2.4)” to correct for batch effects across different samples. PCA was performed on the integrated data, and the top 20 principal components were selected for downstream dimensionality reduction and clustering based on the elbow plot. Unsupervised clustering and visualization of cell subpopulations were performed through a combination of t-SNE dimensionality reduction and the Louvain algorithm. For cell type annotation, we employed a two-step approach. First, unsupervised clustering was performed using the Louvain algorithm implemented in Seurat with a resolution parameter of 0.5. Second, the resulting clusters were annotated using the “SingleR (version 2.12.0)” package with reference to the Human Primary Cell Atlas database (https://www.humancellatlas.org/; last accessed: 12 December 2025). To validate the annotations, we examined the expression of canonical marker genes for each assigned cell type (e.g., *PTPRC* for immune cells, *EPCAM* for epithelial cells, *PECAM1* for endothelial cells) and confirmed that the annotated clusters showed the expected marker expression patterns. To quantitatively assess cell-type-specific expression patterns while accounting for donor-to-donor variability and the sparse nature of single-cell data, we applied a mixed-effects model using the “MAST (version 1.36.0)” package. For each core gene, we modeled expression level as a function of cell type, with donor included as a random intercept to control for inter-donor variability. The model was specified as: expression ~ cell_type + (1 | donor), where expression was modeled using a hurdle model to account for the bimodal distribution typical of scRNA-seq data (dropout events and continuous expression). Genes with a FDR-adjusted *p*-value < 0.05 were considered significantly differentially expressed between cell types. This approach ensures that the reported cell-type-specific expression patterns are not driven by donor-specific effects or technical dropout artifacts. Ultimately, the analysis of the expression distribution of core target genes across each cell type systematically revealed the cell-specific expression patterns of these genes within the ccRCC microenvironment.

### 4.12. Molecular Docking

To elucidate the molecular interaction mechanism between OTA and core target gene proteins, this study employed molecular docking techniques. The 3D structure of OTA in SDF format, Section 4.1, served as the ligand. Three-dimensional crystal structures of the five core target proteins were obtained from the RCSB Protein Data Bank (PDB) database (https://www.rcsb.org/pages/about-us/index; last accessed: 12 December 2025): IGFBP3 (PDB ID: 7WRQ, chain A), ITGA5 (PDB ID: 4WJK, chain A), PYGL (PDB ID: 3CEJ, chain A, B), and LTB4R (PDB ID: 7K15, chain A). For SLC22A8, for which no experimental structure is available, the AlphaFold-predicted structure (UniProt ID: Q8TCC7; AlphaFold DB entry: AF-Q8TCC7-F1) was obtained from the AlphaFold Protein Structure Database (https://alphafold.com/; last accessed: 12 December 2025). Structural preprocessing of the receptor proteins involved the removal of water molecules, proto-ligands, and unnecessary cofactors, along with the addition of hydrogen atoms and Gasteiger charges using PyMol 2.6.0 software (Schrödinger, LLC, New York, NY, USA). The ligand OTA was prepared in AutoDockTools 1.5.7 software (The Scripps Research Institute, La Jolla, CA, USA), with torsional bonds set as rotatable. Subsequently, the spatial dimensions of the docking box were defined based on the center coordinates of the receptor protein’s active pocket, taking into account the spatial dimensions of the ligand molecules and the volume parameters of the pocket. For IGFBP3, the binding pocket was centered on the IGF-1 binding region (coordinates from PDB 7WRQ); for ITGA5, the RGD-binding pocket in the β-propeller domain was used; for PYGL, the glycogen-binding site was selected; for SLC22A8, the central cavity of the AlphaFold model was targeted; for LTB4R, the orthosteric ligand-binding pocket was defined using co-crystallized ligand information from PDB 7K15. Grid boxes were set to 25 × 25 × 25 Å with 1.0 Å spacing, covering the entire binding site. Molecular docking simulations were then conducted using AutoDock Vina 1.1.2 software (The Scripps Research Institute, La Jolla, CA, USA), with exhaustiveness set to 8 and up to nine binding modes generated. The scoring function used was the default AutoDock Vina empirical force field, which combines steric, electrostatic, and hydrophobic terms, applying a binding energy threshold of ≤−5.0 kcal/mol to identify stable binding between the receptor and the ligand. Finally, the conformations exhibiting optimal binding energies from the docking results were imported into PyMol software for visualization, emphasizing the binding sites of the ligand molecules on the target proteins and the key types of interactions, such as hydrogen bonding. The RMSD between the docked and crystallographic poses was calculated, with values below 2.0 Å considered acceptable.

## 5. Conclusions

In this study, we employed multiple bioinformatics analysis methods to construct a comprehensive prediction model aimed at systematically investigating the potential molecular mechanisms underlying the development of OTA (a food contaminant)-associated ccRCC. The results indicated that OTA may play a role in the ccRCC process by regulating specific genes and signaling pathways, which subsequently influence cell growth signaling, ECM remodeling, immune evasion, metabolic reprogramming, chronic inflammation, and renal detoxification functions. Molecular docking analysis further demonstrated that OTA exhibited strong binding affinity to all five core target proteins identified, providing additional computational support for the predicted interactions. These findings suggest potential molecular pathways through which OTA might influence ccRCC development; however, experimental validation is required to confirm these mechanistic hypotheses. Future research should concentrate on elucidating the quantitative relationship between the dose and duration of OTA exposure and the risk of ccRCC development. Additionally, it is essential to validate the biological functions and clinical significance of the core genes through standardized in vivo and ex vivo experiments, as well as clinical samples, to enhance clinical translatability and inform the development of treatment protocols for OTA-related ccRCC. Orthogonal computational approaches, such as molecular dynamics simulations or binding free energy calculations (e.g., MM/GBSA), could further strengthen these predictions and are recommended for future studies. Furthermore, future studies could incorporate specificity checks using decoy ligands and decoy proteins to further validate the selectivity of the predicted OTA–target interactions.

## Figures and Tables

**Table 1:** Binding energies of ligand and receptor.

Ligand	Receptor	Binding Energy (kcal/mol)
OTA	IGFBP3	−8.0
OTA	ITGA5	−9.2
OTA	PYGL	−10.5
OTA	SLC22A8	−9.8
OTA	LTB4R	−9.8

**Figure A1:**
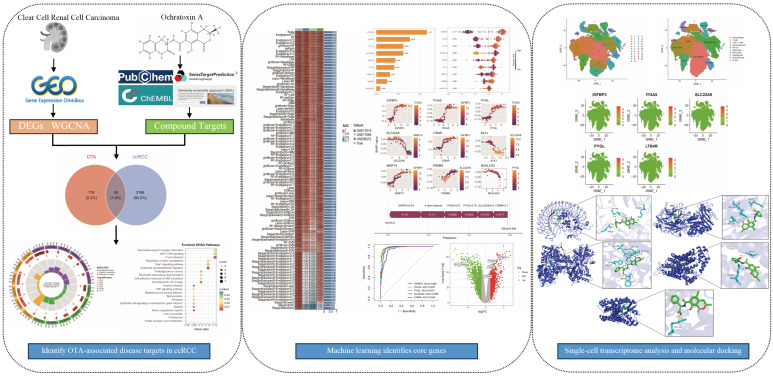
Schematic workflow of the study.

## Data Availability

The original contributions of this study are included in the article and Supplementary Materials. For further inquiries, contact the corresponding author. The custom R scripts used for the analyses in this study are available from the corresponding author upon reasonable request.

## Supplementary Materials

The following supporting information can be downloaded at: https://www.mdpi.com/article/10.3390/ijms27072971/s1.

## Appendix A. Flowchart of Dataset Analysis in This Paper

Figure A1 displays a flowchart illustrating the identification of OTA-associated disease targets in clear cell renal cell carcinoma (ccRCC). First, ccRCC-related genes were obtained from GEO datasets through DEGs and weighted gene co-expression network analysis (WGCNA). Meanwhile, OTA target genes were predicted from public databases (ChEMBL, SEA, SwissTargetPrediction) using its structure retrieved from PubChem. The intersection of these two gene sets yielded OTA–ccRCC common targets. Next, machine learning was employed to screen core target genes, followed by SHAP analysis, single-cell transcriptome analysis, and molecular docking validation to further characterize and verify the core targets.

## References

[1] Ben Miri Y., Benabdallah A., Chentir I., Djenane D., Luvisi A., De Bellis L. (2024). Comprehensive Insights into Ochratoxin A: Occurrence, Analysis, and Control Strategies. Foods.

[2] Li X., Ma W., Ma Z., Zhang Q., Li H. (2022). Recent progress in determination of ochratoxin a in foods by chromatographic and mass spectrometry methods. Crit. Rev. Food Sci. Nutr..

[3] Mubarik Y., Boyetey S.T., Aikins A.R., Mutocheluh M. (2025). Effect of Ochratoxin A (OTA) on the Immune System: A Systematic Review. Toxins.

[4] Wang G., Li E., Gallo A., Perrone G., Varga E., Ma J., Yang B., Tai B., Xing F. (2023). Impact of environmental factors on ochratoxin A: From natural occurrence to control strategy. Environ. Pollut..

[5] Latham R.L., Boyle J.T., Barbano A., Loveman W.G., Brown N.A. (2023). Diverse mycotoxin threats to safe food and feed cereals. Essays Biochem..

[6] Wei G., Guo X., Liang Y., Liu C., Zhang G., Liang C., Huang Z., Zheng Y., Chen S., Dong L. (2023). Occurrence of fungi and mycotoxins in herbal medicines and rapid detection of toxin-producing fungi. Environ. Pollut..

[7] Marcelão C.V.P., Souza M.C., Silva J.J., Couto F.A., Lacorte G.A., Pinto U.M., Maffei J.T., Zacarchenco P.B., Iamanaka B.T., Taniwaki M.H. (2024). Unveiling ochratoxin A and ochratoxigenic fungi in Brazilian artisanal Cheeses: Insights from production to consumption. Food Res. Int..

[8] Więckowska M., Cichon N., Szelenberger R., Gorniak L., Bijak M. (2024). Ochratoxin A and Its Role in Cancer Development: A Comprehensive Review. Cancers.

[9] Zhu X., Al-Danakh A., Zhang L., Sun X., Jian Y., Wu H., Feng D., Wang S., Yang D. (2022). Glycosylation in Renal Cell Carcinoma: Mechanisms and Clinical Implications. Cells.

[10] Zhai K., Dong Z., Geng B., Li Q., Liu Z., Wang D., Chen H., Cui Y. (2025). Identification of biomarkers for renal cell carcinoma in plasma samples measured using liquid chromatography-mass spectrometry and gas chromatography-mass spectrometry. Sci. Rep..

[11] Ma F., Wang S., Xu L., Huang W., Shi G., Sun Z., Cai W., Wu Z., Huang Y., Meng J. (2024). Single-cell profiling of the microenvironment in human bone metastatic renal cell carcinoma. Commun. Biol..

[12] Hsieh J.J., Purdue M.P., Signoretti S., Swanton C., Albiges L., Schmidinger M., Heng D.Y., Larkin J., Ficarra V. (2017). Renal cell carcinoma. Nat. Rev. Dis. Primers.

[13] Li Y., Lih T.M., Dhanasekaran S.M., Mannan R., Chen L., Cieslik M., Wu Y., Lu R.J., Clark D.J., Kołodziejczak I. (2023). Histopathologic and proteogenomic heterogeneity reveals features of clear cell renal cell carcinoma aggressiveness. Cancer Cell.

[14] EAU Renal Cell Cancer Guidelines Panel (2025). EAU Guidelines on Renal Cell Carcinoma 2025—Pocket Guidelines. https://uroweb.org/guidelines/renal-cell-carcinoma.

[15] Powles T., Albiges L., Bex A., Comperat E., Grünwald V., Kanesvaran R., Kitamura H., McKay R., Porta C., Procopio G. (2024). Renal cell carcinoma: ESMO Clinical Practice Guideline for diagnosis, treatment and follow-up. Ann. Oncol..

[16] Zhang Q., Ren H., Ge L., Zhang W., Song F., Huang P. (2023). A review on the role of long non-coding RNA and microRNA network in clear cell renal cell carcinoma and its tumor microenvironment. Cancer Cell Int..

[17] Ye S., Xiang J., Zhou S., Ge Q., Anwaier A., Chang K., Wei G., Lu J., Tian X., Zhu S. (2025). Polystyrene Microplastics Exposure Aggravates Clear Cell Renal Cell Carcinoma Progression via the NF-κB and TGF-β Signaling Pathways. Adv. Sci..

[18] Claeys L., De Saeger S., Scelo G., Biessy C., Casagrande C., Nicolas G., Korenjak M., Fervers B., Heath A.K., Krogh V. (2022). Mycotoxin Exposure and Renal Cell Carcinoma Risk: An Association Study in the EPIC European Cohort. Nutrients.

[19] Polovic M., Dittmar S., Hennemeier I., Humpf H.U., Seliger B., Fornara P., Theil G., Azinovic P., Nolze A., Köhn M. (2018). Identification of a novel lncRNA induced by the nephrotoxin ochratoxin A and expressed in human renal tumor tissue. Cell Mol. Life Sci..

[20] Fahmy N., Woo M., Alameldin M., Macdonald K., Goneau L.W., Cadieux P., Pautler S.E. (2014). Ochratoxin A is not detectable in renal and testicular tumours. Can. Urol. Assoc. J..

[21] Bendele A.M., Carlton W.W., Krogh P., Lillehoj E.B. (1985). Ochratoxin A carcinogenesis in the (C57BL/6J X C3H)F1 mouse. J. Natl. Cancer Inst..

[22] Stoev S.D. (2020). Long term preliminary studies on toxic and carcinogenic effect of individual or simultaneous exposure to ochratoxin A and penicillic acid in mice. Toxicon.

[23] Zheng J., Zhang Y., Xu W., Luo Y., Hao J., Shen X.L., Yang X., Li X., Huang K. (2013). Zinc protects HepG2 cells against the oxidative damage and DNA damage induced by ochratoxin A. Toxicol. Appl. Pharmacol..

[24] Liang R., Shen X.L., Zhang B., Li Y., Xu W., Zhao C., Luo Y., Huang K. (2015). Apoptosis signal-regulating kinase 1 promotes Ochratoxin A-induced renal cytotoxicity. Sci. Rep..

[25] Arbillaga L., Azqueta A., Ezpeleta O., López de Cerain A. (2007). Oxidative DNA damage induced by Ochratoxin A in the HK-2 human kidney cell line: Evidence of the relationship with cytotoxicity. Mutagenesis.

[26] Özcan Z., Gül G., Yaman I. (2015). Ochratoxin A activates opposing c-MET/PI3K/Akt and MAPK/ERK 1-2 pathways in human proximal tubule HK-2 cells. Arch. Toxicol..

[27] Darbuka E., Gürkaşlar C., Yaman I. (2021). Ochratoxin A induces ERK1/2 phosphorylation-dependent apoptosis through NF-κB/ERK axis in human proximal tubule HK-2 cell line. Toxicon.

[28] Zhu L., Zhang B., Dai Y., Li H., Xu W. (2017). A Review: Epigenetic Mechanism in Ochratoxin A Toxicity Studies. Toxins.

[29] Hodges S.L., Bouza A.A., Isom L.L. (2022). Therapeutic Potential of Targeting Regulated Intramembrane Proteolysis Mechanisms of Voltage-Gated Ion Channel Subunits and Cell Adhesion Molecules. Pharmacol. Rev..

[30] Su X., Chen D., Zhu L., Jia H., Cai J., Li P., Han B., Wang D., Li H., Fan J. (2022). SGSM2 inhibits thyroid cancer progression by activating RAP1 and enhancing competitive RAS inhibition. Cell Death Dis..

[31] Grimberg A. (2000). P53 and IGFBP-3: Apoptosis and cancer protection. Mol. Genet. Metab..

[32] Liu Y., Lv H., Li X., Liu J., Chen S., Chen Y., Jin Y., An R., Yu S., Wang Z. (2021). Cyclovirobuxine inhibits the progression of clear cell renal cell carcinoma by suppressing the IGFBP3-AKT/STAT3/MAPK-Snail signalling pathway. Int. J. Biol. Sci..

[33] Xiao Y., Tao P., Zhang K., Chen L., Lv J., Chen Z., He L., Jia H., Sun J., Cao M. (2024). Myofibroblast-derived extracellular vesicles facilitate cancer stemness of hepatocellular carcinoma via transferring ITGA5 to tumor cells. Mol. Cancer.

[34] Wang J.F., Chen Y.Y., Zhang S.W., Zhao K., Qiu Y., Wang Y., Wang J.C., Yu Z., Li B.P., Wang Z. (2022). ITGA5 Promotes Tumor Progression through the Activation of the FAK/AKT Signaling Pathway in Human Gastric Cancer. Oxid. Med. Cell. Longev..

[35] Che X., Tian X., Wang Z., Zhu S., Ye S., Wang Y., Chen Y., Huang Y., Anwaier A., Yao P. (2024). Systematic multiomics analysis and in vitro experiments suggest that ITGA5 could serve as a promising therapeutic target for ccRCC. Cancer Cell Int..

[36] Ji Q., Li H., Cai Z., Yuan X., Pu X., Huang Y., Fu S., Chu L., Jiang C., Xue J. (2023). PYGL-mediated glucose metabolism reprogramming promotes EMT phenotype and metastasis of pancreatic cancer. Int. J. Biol. Sci..

[37] Li M., Zhu G., Liu Y., Li X., Zhou Y., Li C., Wang M., Zhang J., Wang Z., Tan S. (2024). Integrated genomic and proteomic analyses identify PYGL as a novel experimental therapeutic target for clear cell renal cell carcinoma. Heliyon.

[38] Nigam S.K. (2018). The SLC22 Transporter Family: A Paradigm for the Impact of Drug Transporters on Metabolic Pathways, Signaling, and Disease. Annu. Rev. Pharmacol. Toxicol..

[39] Cha S.H., Sekine T., Fukushima J.I., Kanai Y., Kobayashi Y., Goya T., Endou H. (2001). Identification and characterization of human organic anion transporter 3 expressing predominantly in the kidney. Mol. Pharmacol..

[40] Chen H. (2025). P142: The expression profile of SLC22A6 and SLC22A8 genes in kidney renal clear cell carcinoma and their association with clinical outcomes. Genet. Med. Open.

[41] Long S., Ji S., Xiao K., Xue P., Zhu S. (2021). Prognostic and immunological value of LTB4R in pan-cancer. Math. Biosci. Eng..

[42] Wu H.H., Yan X., Chen Z., Du G.W., Bai X.J., Tuoheti K., Liu T.Z. (2021). GNRH1 and LTB4R might be novel immune-related prognostic biomarkers in clear cell renal cell carcinoma (ccRCC). Cancer Cell Int..

[43] Zhang N., Chen W., Gan Z., Abudurexiti A., Hu X., Sang W. (2020). Identification of biomarkers of clear cell renal cell carcinoma by bioinformatics analysis. Medicine.

[44] Zhang R., Wang R., Zhai S., Shen C., An Y., Liu Q. (2025). Bioinformatic analysis and experimental validation of hub autophagy-related genes as novel biomarkers for type 2 diabetes mellitus and Alzheimer’s disease. PeerJ.

[45] Solano-Aguilar G., Molokin A., Botelho C., Fiorino A.M., Vinyard B., Li R., Hibberd P.L. (2016). Transcriptomic Profile of Whole Blood Cells from Elderly Subjects Fed Probiotic Bacteria Lactobacillus rhamnosus GG ATCC 53103 (LGG) in a Phase I Open Label Study. PLoS ONE.

